# ACG-SFE: Adaptive cluster-guided simple, fast, and efficient feature selection for high-dimensional microarray data in binary classification

**DOI:** 10.1371/journal.pone.0331089

**Published:** 2025-09-08

**Authors:** Yi Wei Tye, XinYing Chew, Umi Kalsom Yusof, Samat Tulpar

**Affiliations:** 1 School of Computer Sciences, Universiti Sains Malaysia, Gelugor, Penang, Malaysia; 2 School of Computing and Informatics, Albukhary International University, Alor Setar, Kedah, Malaysia; 3 Smart Manufacturing and Artificial Intelligence, Micron Memory Malaysia Sdn. Bhd., Batu Kawan, Penang, Malaysia; Tecnológico de Monterrey: Tecnologico de Monterrey, MEXICO

## Abstract

Advances in data collection have resulted in an exponential growth of high-dimensional microarray datasets for binary classification in bioinformatics and medical diagnostics. These datasets generally possess many features but relatively few samples, resulting in challenges associated with the “curse of dimensionality”, such as feature redundancy and an elevated risk of overfitting. While traditional feature selection approaches, such as filter-based and wrapper-based approaches, can help to reduce dimensionality, they often struggle to capture feature interactions while adequately preserving model generalization. Therefore, this paper introduces the Adaptive Cluster-Guided Simple, Fast, and Efficient (ACG-SFE) feature selection, a hybrid approach designed to address the challenges of high-dimensional microarray data in binary classification. ACG-SFE enhances the Simple, Fast, and Efficient (SFE) evolutionary feature selection model by integrating hierarchical clustering to dynamically group correlated features based on the optimal number of clusters determined by the Silhouette index, Davies-Bouldin score, and the feature-to-observation ratio while adaptively selecting representative features within clusters using mutual information and adjusting the selection threshold through a progress factor. This hybrid filter-wrapper approach improves feature interactions, effectively minimizing redundancy and overfitting while enhancing classification performance. The proposed model is assessed against four state-of-the-art evolutionary feature selection models on 11 high-dimensional microarray datasets. Experimental results indicate that ACG-SFE effectively selects a small yet pertinent feature subset, minimizing redundancy while attaining enhanced classification accuracy and F-measure. Furthermore, its reduced RMSE between train and test accuracy substantiates its capability to reduce overfitting, outperforming comparative models. These findings establish ACG-SFE as an effective feature selection model for handling high-dimensional microarray data in binary classification, enhancing classification accuracy while selecting minimal relevant features to reduce unnecessary complexity and the risk of overfitting.

## Introduction

Microarray datasets, typically generated through advanced omics technologies, such as genomics and transcriptomics [1], enable simultaneous measurement of thousands of gene expressions, making them valuable for discovering biomarkers and improving medical diagnostics [2]. However, microarray data is extremely high-dimensional, typically containing thousands or even tens of thousands of features (genes) but only a few patient samples [3], which poses significant challenges for binary classification algorithms. Many measured features (genes) in these datasets are irrelevant or noisy, introducing redundancy and unnecessary model complexity, which increases computational cost, heightens the risk of overfitting, and reduces predictive performance in binary classification [4].

Consequently, dimensionality reduction techniques are vital preprocessing methods for addressing challenges in high-dimensional gene expression data, as they compress high-dimensional data into a lower-dimensional form while preserving important information [5]. Generally, dimensionality reduction techniques are categorized into feature extraction and feature selection [6]. Feature extraction transforms the original feature into a lower-dimensional feature space by combining original features, but this transformation often sacrifices the features’ interpretability [7], making it less suitable for medical diagnostics applications where understanding gene relationships is crucial. Conversely, feature selection preserves interpretability by directly selecting the most informative features (genes) while maintaining clear relationships with class labels [8], making it highly suitable for microarray data analysis. Selected genes from microarray data often represent known biomarkers or critical pathways involved in disease progression [9,10], thereby reinforcing the biological relevance and clinical interpretability of feature selection models. Furthermore, feature selection is computationally efficient than feature extraction, as it reduces dimensionality without generating new features [11], making it a preferable approach for high-dimensional microarray data.

Feature selection (FS) techniques are typically categorized into filter, wrapper, and embedded approaches [12]. Filter approaches evaluate features independently using statistical measures, such as correlation and mutual information, offering computational efficiency but ignoring interactions between features and their combined effects with the classifier [12], potentially resulting in suboptimal selections. In contrast, wrapper approaches iteratively evaluate feature subsets based on predictive performance with a specific classifier [13]. Although wrapper approaches implicitly capture beneficial feature interdependencies through this evaluation process, they do not explicitly penalize feature redundancy, which can result in redundant feature subsets [14] and a heightened risk of overfitting, particularly in small-sample datasets [15,16]. Embedded approaches perform feature selection during the model training process, providing adaptive selection but limiting generalizability due to their dependency on specific classifier assumptions [17].

Considering these limitations and guided by the no free lunch theorem that no single model universally outperforms others [18], recent studies have increasingly adopted hybrid filter-wrapper feature selection approaches. These approaches initially apply filter approaches to reduce dimensionality efficiently and subsequently use wrapper approaches to refine feature subsets by capturing feature interactions, ultimately improving classification accuracy and providing more meaningful support for clinical decision-making [19].

Consequently, this paper introduces the Adaptive Cluster-Guided Simple, Fast, and Efficient Feature Selection (ACG-SFE) model, a hybrid feature selection approach designed to address the challenges of high-dimensional microarray data in binary classification. By integrating filter and wrapper approaches, ACG-SFE addresses the limitations of independent evaluation in filter approaches and significantly reduces the risks of redundancy and overfitting associated with wrapper approaches. The model effectively captures feature interactions, minimizes redundancy, and enhances generalization via hierarchical clustering and adaptive cluster regularization.

The key contributions of ACG-SFE are as follows:

- **Dynamic hierarchical clustering-based feature grouping:**

Design a hierarchical clustering technique that dynamically determines the optimal number of feature clusters using Silhouette and Davies-Bouldin scores, along with the feature-to-observation ratio. This technique effectively groups correlated features, reduces redundancy, and enhances feature interactions.

- **Adaptive mutual information-based intra-cluster regularization:**

Introduces an adaptive regularization technique that utilizes mutual information to select informative features within each cluster, dynamically adjusts the selection threshold through a progress factor. This technique prevents excessive feature retention, reduces overfitting, and improves generalization performance.

- **Hybrid filter-wrapper search optimization:**

Integrates dynamic feature clustering and adaptive intra-cluster selection into the Simple, Fast, and Efficient (SFE) heuristic wrapper search. This integration effectively identifies an optimal feature subset, significantly enhancing model accuracy while minimizing feature redundancy.

The remainder of this paper is organized as follows: The related works section reviews related work on filter, wrapper, and hybrid feature selection techniques for high-dimensional microarray data. The research method section presents the ACG-SFE model, detailing its methodology and key components. The experiment design section describes the experimental configuration, including datasets, benchmark models, and evaluation metrics. The results and discussion section presents the results and discusses ACG-SFE’s effectiveness in reducing redundancy, mitigating overfitting, and optimizing feature selection. Finally, the conclusion summarizes key findings and suggests potential future research directions.

### Related works

#### Filter feature selection.

Filter feature selection approaches evaluate each feature’s relevance to the target independently, using statistical or information-theoretic measures [20]. This characteristic makes them computationally efficient and less prone to overfitting in high-dimensional microarray datasets since they do not involve training a classifier during selection [21]. Common filter techniques rank genes by metrics such as t-test scores, chi-square values, correlation, or Mutual Information (MI), selecting the top-ranked genes as potential biomarkers [22]. Correlation-based filter approaches commonly employ Pearson’s correlation to rank features based on linear relationships [23], while Spearman’s correlation detects monotonic relationships [24]. However, strong correlations among selected features introduce redundancy, negatively impacting model performance and increasing the risk of overfitting. To address this redundancy, advanced correlation-based methods like Resampling Fast Correlation-Based Filter (RFCBF) simultaneously evaluate feature-class relevance and redundancy between features, using resampling to stabilize feature selection and improve accuracy [25].

Unlike correlation, MI measures the dependency between features based on information theory [26], effectively capturing both linear and nonlinear relationships between features and class labels [27]. For instance, Zhang et al. introduced a Maximum Conditional Mutual Information (MCMI)-based filter method, effectively reducing redundancy in high-dimensional microarray datasets by selecting genes with strong predictive power and minimal redundancy, thus enhancing classification accuracy and model simplicity [28]. Additionally, Morán-Fernández et al. proposed a low-precision MI-based feature selection approach optimized for devices with limited computational capabilities, demonstrating that reducing the numeric precision of calculations (to 16-bit or even 8-bit) can maintain high classification accuracy on microarray datasets while significantly decreasing computational requirements [29].

In addition to these metrics-based methods, clustering techniques have recently gained prominence for their effectiveness in addressing redundancy by grouping correlated features and selecting representative ones from each cluster [30,31]. For instance, Asghari et al. introduced the Best Clustering Normalized Mutual Information Quantile (BC-NMIQ) model, combining clustering with mutual information ranking and using the Incremental Association Markov Blanket (IAMB) algorithm to select optimal subsets, resulting in enhanced classification accuracy on high-dimensional medical datasets [32]. Similarly, Guo proposed the K-means Neighbourhood Component Feature Selection (KNCFS), which employs correlation-based clustering to group collinear features, subsequently constructing subspaces with lower inter-feature correlations to select robust and informative feature subsets in high-dimensional datasets effectively [33]. These filter and cluster-based approaches have the advantage of speed and can handle ultra-high dimensions, but they struggle to fully capture complex interactions between selected features and the classifier since they do not directly consider a classifier’s feedback.

#### Wrapper-based feature selection.

Wrapper methods approach feature selection as an optimization problem guided by a predictive model. Instead of ranking features by a metrics score, wrappers repeatedly evaluate candidate feature subsets using a classifier to directly assess subset quality in terms of model performance [13]. This strategy allows wrappers to consider feature interactions and their joint contribution to classification accuracy. Techniques like Recursive Feature Elimination (RFE) and Exhaustive Feature Selection (EFS) were early wrappers used for feature selection, often wrapping KNN, SVM, or other classifiers to search for the best subset [34,35]. By exploring combinations of features, wrapper methods can achieve higher predictive accuracy than filters; however, this advantage comes at a substantially increased computational cost [15,16].

Exhaustively searching all subsets is infeasible for tens of thousands of features in microarray data, prompting researchers to adopt Evolutionary Computation (EC)-based techniques, such as Genetic Algorithms (GA), Binary Particle Swarm Optimization (BPSO), Binary Differential Evolution (BDE), and single-agent heuristics like Simple, Fast, and Efficient (SFE) to effectively explore large search spaces and select informative features with strong predictive performance [36,37]. For instance, Krishna and Rajarajeswari proposed Mutual Fuzzy Swarm Optimization (MFSO), which integrates MI, fuzzy logic, and PSO to select informative and biologically meaningful features effectively, significantly enhancing diagnostic accuracy across multiple microarray datasets [38]. Similarly, Li et al. developed a two-stage hybrid biomarker selection approach combining ensemble filtering and BDE enhanced by binary African vultures optimization (EF-BDBA), demonstrating superior biomarker selection performance by balancing exploration and exploitation effectively during optimization [39].

Recent studies have introduced computationally efficient wrapper-based algorithms to address the computational intensity of traditional evolutionary methods. One prominent example is the Simple, Fast, and Efficient (SFE) algorithm, which utilizes a single-agent heuristic to prune redundant or irrelevant features in a non-selection operator quickly. It then identifies informative features through a selection operator, selecting smaller yet highly informative feature subsets than population-based methods [36]. An enhanced variant, SFE-PSO, further incorporates PSO strategies, balancing rapid convergence and comprehensive search capability [36]. However, despite these strengths, SFE and its variants still face challenges such as premature convergence and susceptibility to overfitting, particularly due to their reliance on validation accuracy and static selection criteria, which can negatively impact their generalization to new data.

#### Hybrid feature selection.

Given the complementary strengths of filters and wrappers, hybrid feature selection approaches have emerged as a popular strategy for high-dimensional microarray data [40]. Hybrid approaches typically follow a two-stage or multi-stage selection process where a fast filter approach first drastically reduces dimensionality by performing coarse screening of the features, followed by a wrapper approach that refines the selected subset by capturing feature interactions [40]. Recent studies have demonstrated that such hybrid approaches can achieve high accuracy with fewer features than pure filters or wrappers alone.

For instance, Song et al. introduced a three-phase hybrid feature selection called Correlation-guided clustering with PSO (HFS-C-P), which first employs a filter-based method to eliminate irrelevant features rapidly, then clustering correlated features efficiently using correlation-guided clustering, and finally refining the selected feature subsets through an improved BPSO that incorporates relevance-guided swarm initialization and adaptive disturbance strategies, achieving a strong balance between computational efficiency and classification accuracy [41]. Similarly, Anosh Babu et al. proposed a two-stage clustering-based hybrid feature selection model combining k-means clustering and a signal-to-noise ratio filter to eliminate redundancy and noise, subsequently applying Cellular learning automata combined with Ant Colony Optimization (CLACO) as a wrapper to pinpoint highly discriminative genes, resulting in improved performance on cancer microarray datasets [42]. Pirgazi et al. proposed a two-phase hybrid method integrating Relief filtering for initial feature weighting with the Shuffled Frog Leaping Algorithm (SFLA) and Incremental Wrapper Subset Selection with Replacement (IWSSr) in the wrapper phase, effectively identifying compact and highly predictive gene subsets from high-dimensional gene expression data [43].

While these hybrid feature selection methods effectively balance computational efficiency and classification accuracy, they often lack adaptability due to their sequential design. Once the features are removed in the filtering step, they are typically not reconsidered, potentially causing the premature discard of valuable features. Conversely, retaining redundant or irrelevant features heightens the risk of overfitting during wrapper-based optimization. Thus, there is a need for an adaptive hybrid model capable of dynamically coordinating filter and wrapper approaches to select optimal, non-redundant subsets, thereby reducing overfitting and ultimately improving generalization performance on high-dimensional microarray data.

### Research method

This paper proposes the Adaptive Cluster-Guided Simple, Fast, and Efficient Feature Selection (ACG-SFE) model, which is an advanced feature selection model that builds upon the Simple, Fast, and Efficient (SFE) [36] feature selection model to address the challenges of existing feature selection models highlighted in the related works section. It enhances SFE by introducing dynamic clustering and adaptive regularization filter mechanisms into the wrapper SFE model to preserve valuable features, reduce redundancy, capture important feature interactions, and enhance generalization for high-dimensional microarray data. The following section outlines ACG-SFE and its key components in detail.

### Overview of the ACG-SFE model

**Fig 1:**
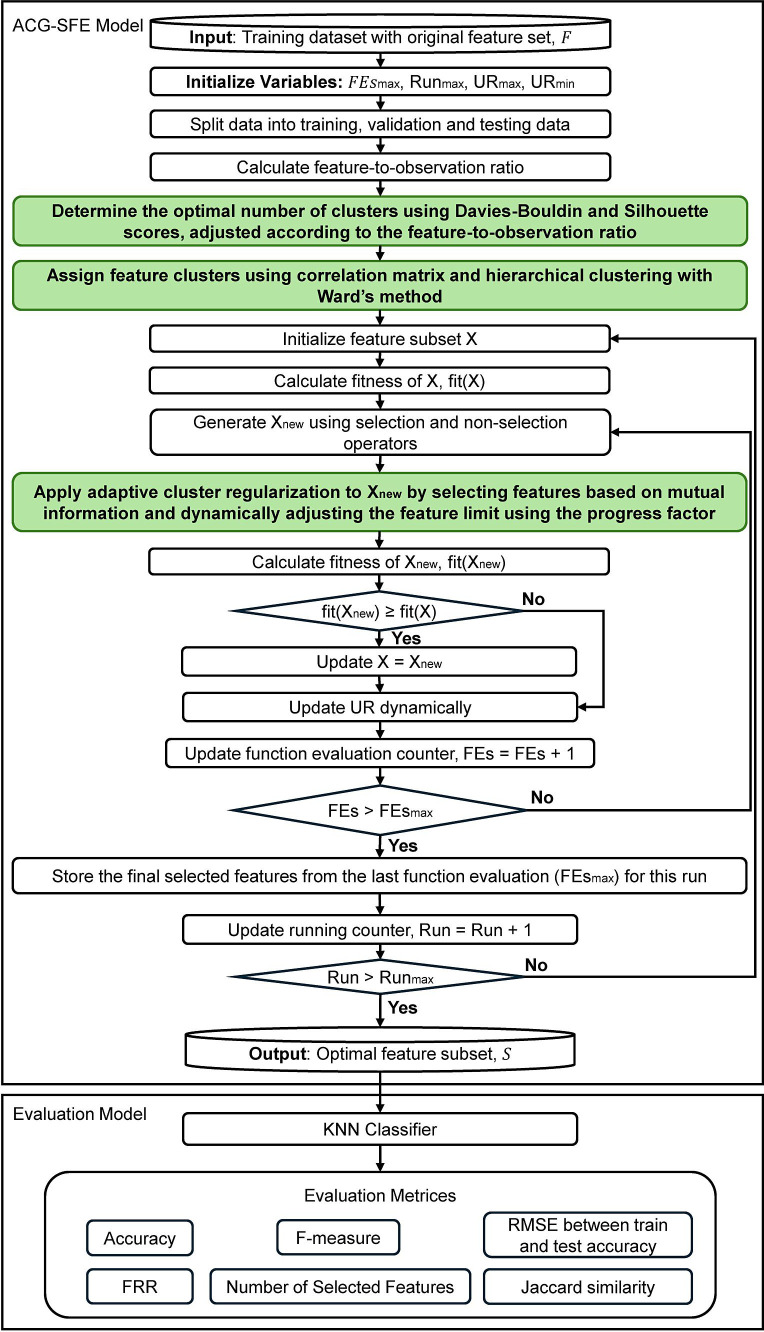
Adaptive Cluster-Guided Simple, Fast, and Efficient (ACG-SFE) model.

Fig 1 illustrates the proposed ACG-SFE model, highlighting the newly introduced components and enhancements over the baseline SFE model [36] in green. ACG-SFE integrates the two phases’ search strategy of the SFE algorithm, initially performing rapid global exploration to remove redundant or irrelevant features, followed by targeted exploitation to reintroduce highly informative features, resulting in a refined initial feature subset [36].

Unlike the standard SFE, which would stop after these two phases, ACG-SFE further refines the feature subset by introducing a dynamic hierarchical clustering that groups correlated features in which its optimal number of clusters is determined using the Davies-Bouldin index and the Silhouette score and dynamically adjusted according to the feature-to-observation ratio. By incorporating feature clusters obtained from hierarchical clustering into the SFE wrapper’s iterative selection process to select representative features from each cluster, ACG-SFE reduces redundancy caused by highly correlated features and enhances interactions between the selected feature subset and the classifier, thus improving overall classification performance.

ACG-SFE also introduces an adaptive cluster regularization mechanism to reduce overfitting by implementing two complementary strategies. First, a mutual information-based selection selects representative and informative features within each cluster, effectively reducing redundancy. Second, an adaptive feature selection threshold, controlled by a progress factor, gradually limits the number of features as optimization progresses. Together, these strategies ensure a compact and generalizable feature subset, minimizing complexity and enhancing the model’s predictive performance on unseen data. The following sections detail each component of the ACG-SFE model.

### Search mechanism

**Fig 2:**
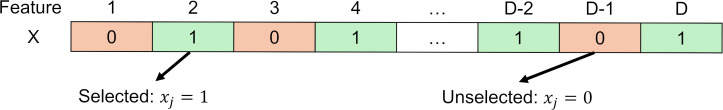
Binary representation of feature subset.

The search mechanism of ACG-SFE operates on a binary-coded feature space, where each feature is represented by a binary state, with 1 indicating a selected feature and 0 indicating an unselected one, as illustrated in Fig 2. Similar to SFE, the initial search process in ACG-SFE consists of two complementary phases: exploration and exploitation, guided by adaptive operators.

#### Exploration (non-selection operator).

In the exploration phase, the non-selection operator (lines 24–29 in Algorithm 1) globally searches the feature space, systematically switching less informative features from selected (1) to non-selected (0) status. The operator uses an adaptive non-selection rate (UR), starting high to remove redundant or irrelevant features aggressively and gradually decreasing as the search progresses [36]. This adaptive adjustment ensures a balanced exploration-exploitation process, refining the feature subset without prematurely discarding valuable features.

#### Exploitation (selection operator).

In the exploitation phase, the selection operator (lines 30–37 in Algorithm 1) locally refines the feature subset by reactivating previously excluded features that significantly enhance model performance. This operator ensures the feature subset never becomes empty, maintaining at least one selected feature to prevent premature convergence and further enhancing the exploitation capability of the model [36].

Algorithm 1 presents the pseudo-code for the proposed ACG-SFE, illustrating the exploration-exploitation operators’ process (lines 24–37 in Algorithm 1) used to rapidly produce an initial feature subset significantly smaller than the original feature set. However, since the features have been evaluated independently, the next section introduces and details the clustering-based approach that captures feature interrelationships to reduce redundancy, enhance generalization, and reduce overfitting.

### Dynamic hierarchical clustering-based feature grouping technique

To effectively manage redundancy and feature interdependence, ACG-SFE employs hierarchical clustering using Ward’s method and Pearson correlation to group correlated features, enabling selection decisions at the cluster level rather than individually. Unlike existing clustering-based FS models, ACG-SFE dynamically determines the optimal number of clusters by evaluating multiple cluster sizes using Davies-Bouldin and Silhouette indices, adjusted based on the dataset’s feature-to-observation (Algorithm 2 and lines 14–15 in Algorithm 1). This dynamic clustering approach ensures that each cluster group strongly correlates features while clearly distinguishing between clusters, simplifying the search space, and preventing redundant evaluation of correlated features.

**Algorithm 1: ACG-SFE feature selection algorithm**

**  Input:** Training dataset with original feature set, 𝐹 ={f_1_,f_2_,f_3_,…,f}

**  Output:** Optimal feature subset, S ={𝑠_1_,𝑠_2_,𝑠_3_,…,𝑑}

1  **Begin:**

2   ***Initialize variables:***

3     𝐹𝐸𝑠_max_: Maximum number of function evaluations

4     Run_max_: Maximum number of run

5     𝑈𝑅_max_: Initial value of non-selection operator rate

6     𝑈𝑅_min_: Final value of non-selection operator rate

7 ***  Split data into training, validation, and testing sets:***

8     Train_Input: Feature matrix for training data

9     Train_Target: Corresponding labels for model training data

10    Fold_Train_Ind: Indices for training data within each cross-validation fold

11    Fold_Test_Ind: Indices for validation data within each cross-validation fold

12    Test_Input: Feature matrix for test data

13    Test_Target: Corresponding labels for the test data

14   Calculate feature-to-observation ratio of Train_Input, feature_obs_ratio = Number of features/ Number of observations

15   Determine num_clusters, the optimal number of clusters, using ***Algorithm 2*** based on feature_obs_ratio

16   feature_clusters = Feature cluster assignments using num_clusters based on ***Algorithm 3***

17   ***While*** Run ≤ Run_max_ ***Do***

18    Initialize feature subset 𝑋={𝑥_1_,𝑥_2_,…,𝑥_D_} randomly in the search space

19    Calculate the fitness of 𝑋 for validation data: fit(X)

20    Calculate the number of features in the dataset, 𝑁𝑣𝑎𝑟

21    𝐹𝐸𝑠 = 1

22    ***While*** 𝐹𝐸𝑠 ≤ 𝐹𝐸𝑠_max_ ***Do***

23     𝑋_𝑁𝑒𝑤 _= 𝑋

24     𝑈𝑅 = 𝑈𝑅_𝑚𝑎𝑥_

25     𝑈𝑁 = [UR × 𝑁𝑣𝑎𝑟] % The number of features to change to non-selected mode

26     U_II𝑑𝑒𝑥 = find the indexes of selected features in 𝑋

27     𝑈 = Generate 𝑈𝑁 random number between 1 to the number of selected features in 𝑋

28     *K* = 𝑖I𝑑𝑒𝑥(𝑈)

29     Set 𝑋_𝑁𝑒𝑤_(*K*) = 0**% non-selection operation**

30 **    If** the number of selected features in 𝑋_𝑁𝑒𝑤_ is ==0

31       𝑋_𝑁𝑒𝑤 _= 𝑋

32       S_Index = find the indexes of non-selected features in X

33       SN = 1**% The number of features to change to selected mode**

34       S = Generate SN random number between 1 to the number of non-selected features in *X*

35       *K*  = 𝑖I𝑑𝑒𝑥(S)

36       Set 𝑋_𝑁𝑒𝑤_(*K*) = 1**% selection operation**

37 **    End if**

38     adaptive_base_features_per_cluster = max(1, ⌈(𝐹𝐸𝑠_max_ − 𝐹𝐸𝑠)/ 𝐹𝐸𝑠_max_ × 3)⌉

39     𝑋_𝑁𝑒𝑤 _= Apply adaptive MI-based intra-cluster regularization to 𝑋_𝑁𝑒𝑤_ based on ***Algorithm 4***

40     Calculate the fitness of 𝑋_𝑁𝑒𝑤_ for validation data: fit(𝑋_𝑁𝑒𝑤_)

41     **If** fit(𝑋_𝑁𝑒𝑤_) ≥ fit(𝑋)

42       𝑋 = 𝑋_𝑁𝑒𝑤_

43       fit(𝑋) = fit(𝑋_𝑁𝑒𝑤_)

44     **End if**

45     𝑈𝑅 = (𝑈𝑅_𝑚𝑎𝑥_ − 𝑈𝑅_𝑚𝑖I_) × ((𝐹𝐸𝑠_max_ – 𝐹𝐸𝑠)/ 𝐹𝐸𝑠_max_) + 𝑈𝑅_𝑚in_

46     𝐹𝐸𝑠 = 𝐹𝐸𝑠 + 1

47 **   End while**

48    FN_run_ = Final number of selected features for this run

49    Run = Run + 1

50   **End while**

51** End**

Selecting an appropriate number of clusters is crucial. If too few clusters are used, unrelated features can be clustered together, reducing the precision of feature selection; conversely, too many clusters can fragment related features, increasing redundancy and computational complexity. To address this, Algorithm 2 dynamically determines the clustering range (minimum and maximum cluster counts) based on the dataset’s feature-to-observation ratio (lines 2–9 in Algorithm 2). Specifically, for datasets with a low feature-to-observation ratio (less than 300), the divisor used to determine the maximum number of clusters is set higher, resulting in fewer clusters to ensure closely related features are grouped accurately without unnecessary splitting, which suits datasets where the numbers of features and observations are relatively balanced. Conversely, for datasets with higher ratios (300 or greater), the divisor is slightly decreased to yield more clusters, preventing unrelated features from being grouped excessively, thus effectively managing redundancy.

**Fig 3:**
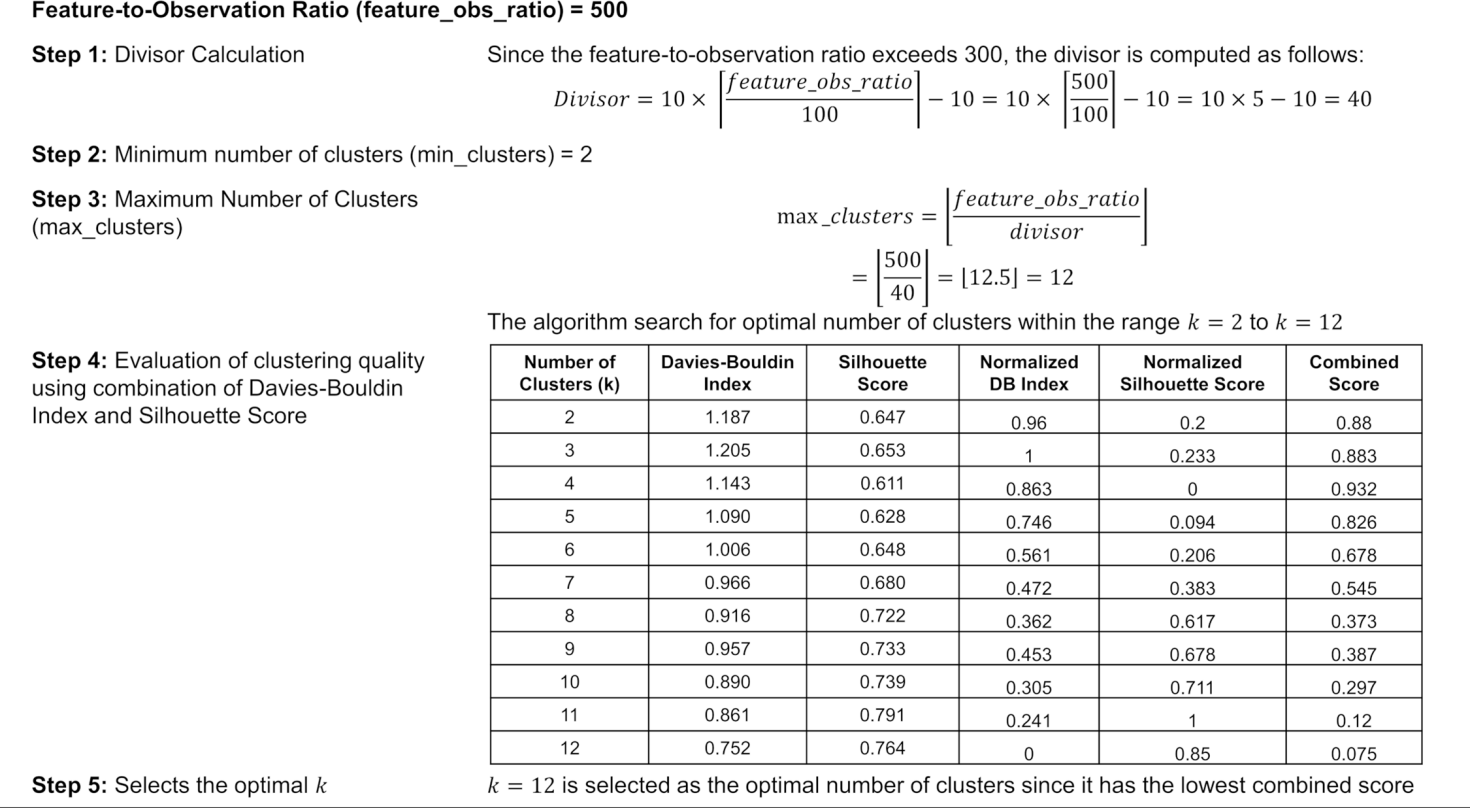
Numerical example for determining the optimal number of clusters in Algorithm 2.

Once the clustering range is defined, each candidate number of clusters within this range is evaluated using the Davies-Bouldin and Silhouette indices (lines 13–23 in Algorithm 2). The Davies-Bouldin index assesses cluster compactness and separation, with lower scores indicating better-defined clusters, while the Silhouette score measures how well features fit within their clusters, with higher scores reflecting more meaningful cluster assignments. Both metrics are normalized via min-max scaling (lines 25–38 in Algorithm 2) to ensure a fair comparison across cluster sizes. The optimal number of clusters is selected based on the lowest combined normalized score, equally weighting both metrics (lines 39–43 in Algorithm 2). For clarity, Fig 3 illustrates this process using Algorithm 2 for training data with a feature-to-observation ratio of 500.

After determining the optimal number of clusters, hierarchical clustering using Ward’s method is applied to group strongly correlated features, minimizing within-cluster variance while maximizing between-cluster separation (Algorithm 3 and lines 16 in Algorithm 1). By conducting feature selection at the cluster level rather than individually, ACG-SFE simplifies the search space, enhances computational efficiency, reduces redundant feature evaluations, and retains the most informative features. The following section describes how these feature clusters are integrated into an adaptive mutual information-based intra-cluster regularization mechanism of ACG-SFE to reduce overfitting.

**Algorithm 2: Determining the optimal number of clusters**

 Input:

 Train_Input: Feature matrix for training data

 feature_obs_ratio: Ratio of the number of features to the number of observations in Train_Input

** Output:** num_clusters: Optimal number of clusters

1 **Begin:**

2  ***Set initial range for clusters:***

3   min_clusters = 2

4 **  If** feature_obs_ratio < 300

5    divisor = 10 × ⌈ feature_obs_ratio/ 100 ⌉

6 **  Else**

7    divisor = 10 × ⌈ feature_obs_ratio/ 100 ⌉ − 10

8   **End if**

9   max_clusters = ⌊ feature_obs_ratio/ divisor ⌋

10  ***Initialize empty arrays to store Davies-Bouldin and Silhouette scores:***

11   db_values = Empty array of size (max_clusters − min_clusters + 1) to store Davies-Bouldin scores

12   sil_values = Empty array of size (max_clusters − min_clusters + 1) to store Silhouette scores

13 ** For** k = min_clusters to max_clusters **Do**

14 **  Try**

15    ***Compute hierarchical clustering using Ward’s method:***

16     Z = Hierarchical clustering linkage matrix using Ward’s method on Train_Input

17     cluster_ids = Cluster labels for each observation through forming k clusters from Z

18    db_values_k_ = Davies-Bouldin index for k clusters to evaluate cluster compactness and separation

19    sil_values_k_ = Mean Silhouette score for k clusters to measure each observation fits within its assigned cluster

20 **  Catch Exception**

21    Set db_values_k_ and sil_values_k_ to NaN

22   **End Try**

23 ** End for**

24  Remove NaN values from db_values and sil_values

25  ***Compute the minimum and maximum Davies-Bouldin and Silhouette scores for normalization:***

26   DB_min_ = Minimum db_values, DB_max_ = Maximum db_values,

    SIL_min_ = Minimum sil_values, SIL_max_ = Maximum sil_values

27 *** Initialize optimal cluster search:***

28   Set best_score = ∞, optimal_clusters = min_clusters

29  **For** k = min_clusters to max_clusters **Do**

30 **  Try**

31 ***   Recompute hierarchical clustering to determine the best k by evaluating normalized scores:***

32     Z = Hierarchical clustering linkage matrix using Ward’s method on Train_Input

33     cluster_ids = Cluster labels for each observation through forming k clusters from Z

34    db_index = Davies-Bouldin index to evaluate cluster compactness and separation

35    sil_score = Mean Silhouette score to measure how well each observation fits within its assigned cluster

36 ***   Normalize Davies-Bouldin and Silhouette scores:***

37     normalized_db = (db_index − DB_min_)/ (DB_max_ − DB_min_)

38     normalized_sil = (sil_score − SIL_min_)/ (SIL_max_ − SIL_min_)

39    combined_score = 0.5 × normalized_db + 0.5 × (1 − normalized_sil)

40    **If** combined_score < best_score

41     best_score = combined_score

42     optimal_clusters = k

43 **   End if**

44 **  Catch Exception**

45    Skip k

46 **  End Try**

47 ** End for**

48  Set num_clusters = optimal_clusters

49 **End**

**Algorithm 3: Clustering features based on correlation matrix**

**Input:**

Train_Input: Feature matrix for training data

num_clusters: Optimal number of clusters

**Output:** feature_clusters: Cluster assignments for features

1 **Begin:**

2   corr_matrix = Correlation matrix of Train_Input

3 ***  Perform hierarchical clustering using Ward’s method:***

4     Z = Hierarchical clustering linkage matrix using Ward’s method on corr_matrix

5     feature_clusters = Cluster labels for each feature through forming num_clusters clusters from Z

6** End**

### Adaptive mutual information-based intra-cluster regularization technique

To further optimize feature selection, ACG-SFE employs an adaptive Mutual Information (MI)-based intra-cluster regularization strategy (Algorithm 4) that refines feature selection within clusters through two complementary mechanisms: MI-based feature ranking and adaptive adjustment of the number of selected features.

**Algorithm 4: Adaptive MI-based Intra-cluster Regularization**

**Input:**

  X: Feature selection mask representing features chosen through non-selection and selection operators

  feature_clusters: Cluster assignments for features

  Train_Input: Feature matrix for training data

  Train_Target: Corresponding labels for model training data

  adaptive_base_features_per_cluster: Base limit on the number of selected features per cluster

  𝐹𝐸𝑠: Current number of function evaluations

  𝐹𝐸𝑠_max_: Maximum number of function evaluations

**  Output:** X_Adaptive: Updated feature selection mask

1 **Begin:**

2  Initialize X_Adaptive as a zero vector of the same size as X

3  unique_clusters = Distinct cluster labels from feature_clusters

4  progress_factor = 𝐹𝐸𝑠/ 𝐹𝐸𝑠_max_

5 **  For** each cluster_id in unique_clusters **Do**

6    feature_indices_in_cluster = Indices of features belonging to cluster_id

7    mi_scores = Mutual information values between each feature in feature_indices_in_cluster and Train_Target using ***Algorithm 5***

8  ***  Intra-cluster selection based on mutual information and adaptive feature limit:***

9     adaptive_feature_limit = ⌈adaptive_base_features_per_cluster×(1+(1 − progress_factor)×0.5 × random_value)⌉

10    selected_features = Top-ranked features from feature_indices_in_cluster based on mi_scores, selecting a number of features equal to the minimum of adaptive_feature_limit and the cluster size

11    Update X_Adaptive to include selected_features

12 ** End for**

13** End**

#### Mutual information-based feature ranking.

ACG-SFE ranks features within each cluster based on their MI with the target labels (lines 6–7 in Algorithm 4). MI quantifies the dependency between a feature and the target, enabling features to be ranked by their relevance. Algorithm 5 computes MI scores by first generating and normalizing a joint histogram of feature values and target labels, then summing over all feature-target probability pairs, which also can be formulated by standard MI formulation [44,45]:


$$\begin{array}{c} MI(X,Y)=\sum {\mathrm{\backslash nolimits}}_{x\in X}\sum {\mathrm{\backslash nolimits}}_{y\in Y}P(x,y)\text{log}\left(\frac{P(x,y)}{P(x)P(y)}\right) \end{array}$$ (1)

where P(x,y) represents the joint probability of feature X and target Y, while P(x) and P(y) are their respective marginal probabilities. Features with higher MI scores, indicating stronger associations with the target labels, are prioritized for selection. This approach reduces redundancy by eliminating each cluster’s less informative or highly correlated features.

**Algorithm 5: Mutual Information Calculation**

**Input:**

  X: Feature values

  y: Target labels

** Output:** mi: Mutual information score between X and y

1 **Begin:**

2   joint_hist = Joint histogram of X and y, normalized as probabilities

3   marg_x = Marginal probability distribution of X

4   marg_y = Marginal probability distribution of y

5   expected_joint = marg_x × marg_y

6   Identify valid entries where joint_hist > 0 to avoid log(0)

7   mi = ∑(joint_hist(valid) × log(joint_hist(valid)/ expected_joint(valid)))

8** End**

#### Adaptive feature limit adjustment.

To prevent overfitting and unnecessary complexity, ACG-SFE adaptively limits the maximum number of selected features per cluster using an evolving threshold (lines 9 in Algorithm 4). Initially, the algorithm allows more features per cluster to promote broad exploration. As optimization progresses, the threshold decreases gradually to emphasize targeted refinement (exploitation).

Specifically, the adaptive feature limit is calculated using two components

- **Adaptive base feature limit per cluster** (line 38 in Algorithm 1):

$$\text{adaptive}\_\text{base}\_\text{features}\_\text{per}\_\text{cluster}=\text{max}(\text{1, }\left\lceil \left(\frac{{FEs}_{max}-FEs}{{FEs}_{max}}\right)\times 3\right\rceil )$$ (2)

This formula systematically decreases the upper bound on the number of features selected per cluster as the number of function evaluations (FEs) progresses. Early on, a higher limit encourages extensive exploration, whereas later stages focus on refining the most impactful subset of features.

- **A Scaling Factor (SF)** introduces controlled randomness to balance exploration and exploitation:

SF=1+(1−progress_factor)×0.5×random_value (3)

Here, the progress factor represents the proportion of optimization completed ($FEs/{FEs}_{max}\;$), and the random value is uniformly sampled from [0,1]. At the early optimization stages (low progress factor), the scaling factor is closer to its upper bound (≈1.5). This stage encourages exploration by allowing the selection of more features per cluster and maintaining diversity through controlled randomness, thus preventing the premature exclusion of potentially useful features. As optimization progresses (high progress factor), SF gradually approaches its lower bound (≈1), reducing the number of features selected per cluster. This shift focuses the algorithm on refining the most informative feature subset while preventing excessive feature selection, thus stabilizing feature selection, reducing redundancy, and effectively mitigating overfitting.

Finally, the adaptive feature limit per cluster is calculated by applying the scaling factor to the adaptive base feature limit:


adaptive_feature_limit=⌈adaptive_base_features_per_cluster×SF⌉ (4)

Once the adaptive feature limit is computed, the number of selected features per cluster is constrained by the smaller value between the adaptive limit and the cluster size. If a cluster contains fewer features than the computed limit, all its features are retained; otherwise, only the top-ranked features (based on mutual information) up to the limit are selected. This adaptive mechanism ensures the selection of a compact, stable, and representative feature subset, effectively reducing redundancy and complexity and enhancing generalization.

### Fitness function

After determining the feature subset through adaptive MI-based intra-cluster regularization, the selected features are evaluated using a fitness function to measure their classification performance (line 40 in Algorithm 1). The fitness function employed by ACG-SFE relies on validation accuracy, assessing how effectively the selected subset generalizes to unseen validation data. Specifically, the validation accuracy is calculated as:


$${Acc}_{val}=\frac{TP+TN}{TP+TN+FP+FN}$$ (5)

where TP (True Positive) and TN (True Negative) denote the number of correctly classified positive and negative samples, respectively, while FP (False Positives) and FN (False Negatives) represent misclassified positive and negative samples.

An iterative validation procedure (lines 41–44 in Algorithm 1) continuously evaluates and refines the feature subset. In each iteration, the updated feature subset ($X_{New}$) is assessed on validation data. If $X_{New}$ achieves equal or higher validation accuracy than the previous best subset, it replaces the previous best subset. Otherwise, the previous best subset is retained to avoid performance degradation. This iterative approach ensures the best feature subset remains compact, stable, high-performing, and generalizable, optimizing feature reduction and classification performance across diverse datasets.

### Experiment design

#### Datasets.

Experiments are conducted using 11 high-dimensional real-world microarray datasets, as listed in Table 1, to evaluate the performance and generalization ability of the proposed ACG-SFE algorithm. These datasets are characterized by a high dimensionality, with features ranging from 2,000 to 19,993 and observations from 20 to 253, resulting in feature-to-observation ratios between 32.26 and 631.35. Since this paper specifically addresses high-dimensional feature selection without significant class imbalance, the selected datasets have balanced to slightly imbalanced binary class distributions, with Imbalance Ratios (IR) ranging from 1 to 4.84. All datasets are publicly accessible and sourced from reputable repositories, including Mendeley Data [46], GitHub repositories [47,48], Shenzhen University’s repository [49], and the NCBI Gene Expression Omnibus (GEO) database [50]. These public datasets have been widely utilized in feature selection research, providing a reliable benchmark for fair comparisons with existing methods and reproducibility of the proposed ACG-SFE algorithm.

**Table 1:** List of 11 datasets and their description.

No.	Dataset	#Fea.	#Obs.	#Classes	Majority	Minority	IR	#Fea./#Obs.
1	Colon	2000	62	2	40	22	1.82	32.26
2	DLBCL	5469	77	2	58	19	3.05	71.03
3	Prostate GE	5966	102	2	52	50	1.04	58.49
4	Leukemia	7070	72	2	47	25	1.88	98.19
5	ALLAML	7129	72	2	47	25	1.88	99.01
6	CNS	7129	60	2	38	22	1.73	118.82
7	Prostate Cancer	12627	20	2	10	10	1.00	631.35
8	Ovarian Cancer	15154	253	2	162	91	1.78	59.90
9	SMK_CAN_187	19993	187	2	97	90	1.08	106.91
10	Prostate Tumor	10509	102	2	52	50	1.04	103.03
11	Lung Cancer	12533	181	2	150	31	4.84	69.24

Abbreviations: #Fea., number of features; #Obs., number of observations; IR, imbalance ratio (majority/minority); #Fea./#Obs., feature-to-observation ratio (number of features/number of observations).

### Comparative models and parameter settings

The performance of the proposed ACG-SFE algorithm is evaluated against four state-of-the-art feature selection models: BDE [51], BPSO [52,53], SFE [36], and SFE-PSO [36]. Additionally, a scenario Without Feature Selection (WFS) is included as a baseline to demonstrate the practical impact of applying feature selection. BDE and BPSO are included because they are well-known evolutionary algorithms that are widely used for feature selection in high-dimensional datasets and previously benchmarked against SFE. Besides, the SFE algorithm is selected due to its computational efficiency and lightweight state-of-the-art wrapper-based model, which forms the foundation for the proposed ACG-SFE model. Furthermore, the SFE-PSO hybrid model, which combines SFE and PSO, is included to examine whether the proposed ACG-SFE achieves further improvements beyond existing SFE-based hybridizations.

Hyperparameter settings for all algorithms, summarized in Table 2, follow the baseline SFE paper to ensure consistency and fair comparisons throughout the experiments. The population size for population-based evolutionary algorithms, including BDE, BPSO, and the PSO component of SFE-PSO, is consistently set to 20 for fair comparisons. Specifically, BDE employs a Mutation Factor (F) of 0.8 and a Crossover Rate (CR) of 0.2 to balance exploration and exploitation. Meanwhile, BPSO uses an inertia weight (w) of 1, a cognitive coefficient (c1) of 2, and a social coefficient (c2) of 1.5 to achieve a balanced search strategy, combining each particle’s individual experiences with collective swarm intelligence.

**Table 2:** Hyperparameter settings.

Algorithms	Parameter values
WFS	–
BDE [51]	F = 0.8; CR = 0.2; b = 20
BPSO [52,53]	w = 1; c1 = 2; c2 = 1.5
SFE [36], SFE-PSO [36], ACG-SFE	𝑈𝑅_max_ = 0.3; 𝑈𝑅_min_ = 0.001; SN = 1

Parameters: F, mutation factor; CR, crossover rate; b, population size; w, inertia weight; c1, cognitive coefficient; c2, social coefficient; 𝑈𝑅_max_, maximum non‑selection operator rate; 𝑈𝑅_min_, minimum non‑selection operator rate; SN, fixed selection number.

In contrast, SFE, the SFE component of SFE-PSO, and the proposed ACG-SFE utilize a single-agent heuristic approach, dynamically applying non-selection and selection operators. The non-selection operator employs a linearly decaying non-selection operator rate (UR) to remove redundant features, starting from a maximum of 0.3 to broadly explore feature subsets and gradually decreasing toward 0.001 to emphasize exploitation. Additionally, the selection operator ensures that at least one feature remains selected using a fixed selection number (SN = 1), preventing scenarios where no features are selected.

### Experimental setup

Experiments employ the K-Nearest Neighbors (KNN) classifier with k = 1, selected due to its simplicity [54], non-parametric flexibility [55], and sensitivity to the selected features’ quality. Unlike parametric classifiers that rely on predefined data distributions, KNN makes no distributional assumptions, enabling flexible adaptation to diverse datasets [56]. Since KNN classifies data based on feature-space distances [57], it directly benefits from informative feature subsets and is adversely influenced by irrelevant or redundant features, making it highly effective for evaluating feature selection models.

The datasets are partitioned into training (80%) and testing (20%) sets to evaluate generalization performance. To ensure reproducibility and fair comparison across multiple feature selection models, all experiments utilize the same training-test split and identical stratified 5-fold cross-validation partitions within the training data, consistently generated using a fixed random seed of 42. Within the training set, the 5-fold cross-validation strategy iteratively evaluates validation accuracy at each of the 6,000 function evaluation steps, guiding feature selection toward an optimal subset. The final best-selected feature subset, obtained at the end of these evaluations, is then assessed on the test data to confirm the robustness and generalization capability.

Several metrics comprehensively measure feature selection effectiveness, including test accuracy, F-measure, Root Mean Square Error (RMSE), number of selected features, Feature Reduction Rate (FRR), and Jaccard similarity. Test accuracy measures overall predictive performance, while the F-measure evaluates the balance between precision and recall, capturing robustness across different class distributions. The RMSE between training and test accuracy is computed to quantify overfitting, with a lower RMSE indicating better generalization and reduced overfitting, calculated as:


$$\begin{array}{c} RMSE=\sqrt{\frac{1}{n}\sum {\mathrm{\backslash nolimits}}_{i=1}^{n}{\left({Train\;Accuracy}_{i}-{Test\;Accuracy}_{i}\right)}^{2}} \end{array}$$ (6)

The number of selected features and the FRR measure the degree of dimensionality reduction, reflecting how the model removes redundant or non-informative features while preserving essential information. FRR measures the percentage reduction in dimensionality achieved through feature selection, defined as:


$$FRR=\left(1-\frac{Number\;of\;Selected\;Features}{Total\;Features}\right)\times 100$$ (7)

The Jaccard similarity metric evaluates feature selection stability across multiple runs by measuring the similarity between subsets of selected features. Given two index vectors A and B, representing two selected subsets, the Jaccard similarity is calculated as the ratio of the intersection to the union of these subsets [58], with a higher percentage indicating greater consistency and stability of selected features across independent runs, computed as:


$$Jaccard\;Similarity\;(A,B)=\frac{\left|A\cap B\right|}{\left|A\cup B\right|}=\frac{\left|A\cap B\right|}{\left|A|+|B|-|A\cap B\right|}\times 100$$ (8)

Each feature selection model is executed independently for 30 runs to assess the consistency and reliability of its performance. The performance metrics from these runs are then averaged to provide a stable and representative measure of overall performance. Statistical tests, such as the Wilcoxon signed-rank test and the Friedman test, were used to further validate the statistical significance of the results. The Wilcoxon signed-rank test evaluates statistical significance in pairwise comparisons between proposed ACG-SFE and other models, while the Friedman test ranks models across datasets, ensuring robust comparative analysis. The source code of the proposed ACG-SFE model can be accessed publicly at: https://github.com/yiwei9464/ACG-SFE.git.

All experiments are conducted on a desktop equipped with a 13th Gen Intel(R) Core(TM) i9-13900F processor (2.00 GHz) and 64 GB of RAM, running on a 64-bit Windows 11 Pro system to provide sufficient computational power for handling high-dimensional datasets effectively.

## Results and discussion

This section evaluates the performance and selected features of the proposed ACG-SFE and comparative models using the metrics described in the Experiment setup. The analysis covers classification accuracy, overfitting mitigation, feature redundancy reduction, F-measure, the consistency and clustering behavior of selected features, as well as stability. Computational efficiency, measured as the mean runtime across 30 independent runs, is also assessed to evaluate each model’s feasibility and scalability, as provided in S1 File. Statistical significance of all the results is evaluated using the Wilcoxon signed-rank and Friedman tests.

### Classification accuracy

Classification accuracy is a primary metric for evaluating feature selection performance. Table 3 presents the worst, best, mean, and standard deviation values of test classification accuracy across 30 independent runs. The Wilcoxon signed-rank test (significance level of 0.05) is used to assess statistical significance, with symbols “+” (significantly better), “−” (significantly worse), and “≈” (no significant difference) indicating comparative performance relative to benchmark models. Additionally, the mean Friedman test, averaging model ranks across all datasets, comprehensively evaluates the algorithms’ performance.

**Table 3:** Test accuracy (%) across 30 runs (worst, best, mean, and standard deviation) for six models.

No.	Dataset	Metrics	WFS	BDE	BPSO	SFE	SFE-PSO	Proposed ACG-SFE
1	Colon	Worst	66.67	58.33	58.33	41.67	41.67	75.00
		Best	66.67	66.67	66.67	83.33	83.33	75.00
		Mean	66.67 (+)	61.67 (+)	65.56 (+)	63.61 (+)	64.44 (+)	**75.00**
		Std	0.00	4.15	2.88	12.85	10.48	0.00
2	DLBCL	Worst	86.67	80.00	80.00	66.67	53.33	93.33
		Best	86.67	86.67	86.67	100.00	100.00	93.33
		Mean	86.67 (+)	83.33 (+)	86.22 (+)	83.33 (+)	81.78 (+)	**93.33**
		Std	0.00	3.39	1.69	8.88	11.74	0.00
3	Prostate GE	Worst	85.00	75.00	80.00	65.00	65.00	85.00
		Best	85.00	90.00	90.00	95.00	100.00	85.00
		Mean	**85.00 (≈)**	83.67 (≈)	84.83 (≈)	80.83 (+)	82.5 (≈)	**85.00**
		Std	0.00	3.70	2.45	8.72	8.07	0.00
4	Leukemia	Worst	78.57	71.43	78.57	57.14	64.29	100.00
		Best	78.57	85.71	85.71	92.86	100.00	100.00
		Mean	78.57 (+)	78.10 (+)	78.81 (+)	73.10 (+)	77.86 (+)	**100.00**
		Std	0.00	2.61	1.30	9.51	9.63	0.00
5	ALLAML	Worst	85.71	78.57	85.71	50.00	64.29	92.86
		Best	85.71	85.71	85.71	85.71	92.86	100.00
		Mean	85.71 (+)	85.24 (+)	85.71 (+)	70.48 (+)	77.86 (+)	**98.33**
		Std	0.00	1.81	0.00	8.33	7.58	3.07
6	CNS	Worst	66.67	58.33	66.67	41.67	33.33	75.00
		Best	66.67	75.00	66.67	83.33	83.33	75.00
		Mean	66.67 (+)	66.67 (+)	66.67 (+)	60.56 (+)	63.33 (+)	**75.00**
		Std	0.00	3.79	0.00	11.97	11.91	0.00
7	Prostate Cancer	Worst	75.00	75.00	75.00	25.00	25.00	100.00
		Best	75.00	75.00	75.00	100.00	100.00	100.00
		Mean	75.00 (+)	75.00 (+)	75.00 (+)	66.67 (+)	72.50 (+)	**100.00**
		Std	0.00	0.00	0.00	20.06	21.12	0.00
8	Ovarian Cancer	Worst	94.00	94.00	94.00	94.00	92.00	100.00
		Best	94.00	96.00	94.00	100.00	100.00	100.00
		Mean	94.00 (+)	94.27 (+)	94.00 (+)	97.73 (+)	97.27 (+)	**100.00**
		Std	0.00	0.69	0.00	2.15	2.00	0.00
9	SMK_CAN_187	Worst	59.46	54.05	59.46	43.24	43.24	62.16
		Best	59.46	67.57	64.86	75.68	72.97	62.16
		Mean	59.46 (+)	61.35 (≈)	60.99 (+)	58.83 (+)	58.74 (+)	**62.16**
		Std	0.00	3.56	1.83	7.53	6.92	0.00
10	Prostate Tumor	Worst	85.00	80.00	80.00	60.00	45.00	80.00
		Best	85.00	85.00	85.00	90.00	90.00	85.00
		Mean	**85.00 (≈)**	81.50 (+)	82.83 (+)	77.17 (+)	75.83 (+)	84.67
		Std	0.00	2.33	2.52	8.38	9.57	1.27
11	Lung Cancer	Worst	94.44	94.44	94.44	86.11	88.89	100.00
		Best	94.44	97.22	94.44	100.00	100.00	100.00
		Mean	94.44 (+)	95.28 (+)	94.44 (+)	93.24 (+)	94.17 (+)	**100.00**
		Std	0.00	1.29	0.00	3.47	2.86	0.00
**Wilcoxon Test (+| ≈ |-)**			9|2|0	9|2|0	10|1|0	11|0|0	10|1|0	**–**
**Friedman Test (Mean Rank)**			3.63	3.86	3.67	4.24	4.02	**1.59**

Notes: Accuracy values are percentages. Std, standard deviation. Symbols indicate Wilcoxon signed‑rank tests (α = 0.05) comparing ACG‑SFE with each benchmark model: + , ACG‑SFE significantly higher accuracy; − , ACG‑SFE significantly lower accuracy; ≈ , no significant difference.

The proposed ACG-SFE model outperforms benchmark algorithms in 9 out of 11 datasets, demonstrating its superior capability to select highly informative features and effectively reduce overfitting. Specifically, ACG-SFE achieves perfect accuracy (100%) on the leukemia, prostate cancer, ovarian cancer, and lung cancer datasets. It also significantly improves accuracy on colon, DLBCL, ALLAML, CNS, and SMK_CAN_187 datasets, further highlighting its effectiveness in feature selection.

For the Prostate GE dataset, ACG-SFE achieves the same mean accuracy (85.00%) as the WFS scenario but significantly reduces dimensionality, selecting only 8 out of 5,966 features. In the Prostate Tumor dataset, ACG-SFE attains marginally lower mean accuracy (84.67%) than WFS (85.00%), but this difference is not statistically significant based on the Wilcoxon signed-rank test. Moreover, ACG-SFE significantly reduces dimensionality by selecting just 13 out of 10,509 features. These results demonstrate that ACG-SFE selects a more compact and highly relevant feature subset while maintaining comparable classification performance.

Examining the variability of the feature selection models, SFE and SFE-PSO exhibit higher fluctuations in test accuracy, with standard deviations exceeding 5% in 10 out of 11 datasets, indicating instability in feature selection. In contrast, BDE, BPSO, and ACG-SFE exhibit more consistent performance with a standard deviation of less than 5% across all datasets, demonstrating greater reliability and stability.

The Wilcoxon signed-rank and Friedman test results for test accuracy confirm ACG-SFE’s superior performance, consistently outperforming or performing on par with other comparative models. Additionally, ACG-SFE achieves the best average rank (1.59) in the Friedman test, significantly outperforming the other algorithms.

**Fig 4:**
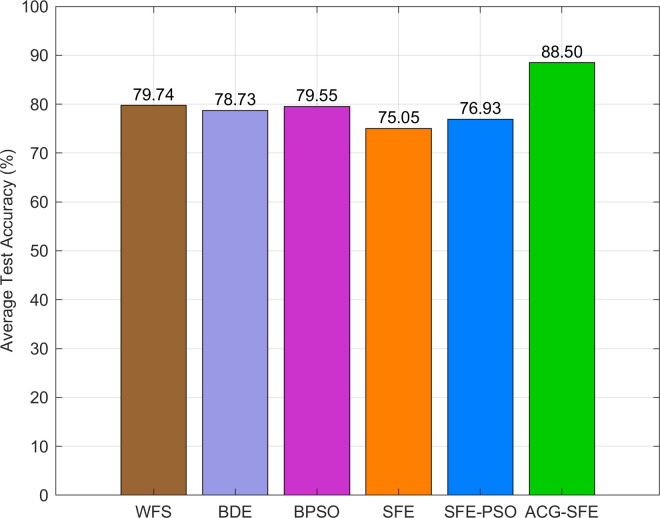
Average test classification accuracy of six feature selection models.

Fig 4 illustrates the average test accuracy across datasets, clearly highlighting ACG-SFE’s superior performance compared to all comparative models. Among the benchmarks, the WFS scenario achieves the highest accuracy, closely followed by BDE and BPSO, indicating their effectiveness in retaining informative features without substantial performance loss. Conversely, SFE and SFE-PSO exhibit lower average accuracy (as illustrated in Fig 4) and higher variability (as shown in Table 3), reflecting instability and potential overfitting.

**Fig 5:**
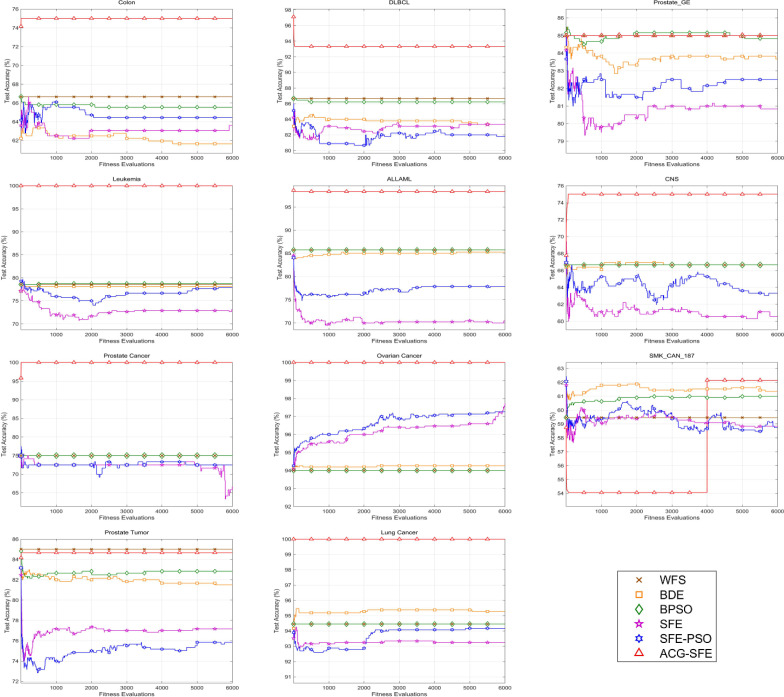
Convergence curve of feature selection algorithms across 11 datasets with KNN classifier.

Fig 5 demonstrates the convergence behaviors of all feature selection models across 6,000 function evaluations. ACG-SFE consistently achieves the highest accuracy across most datasets, including colon, DLBCL, Prostate GE, leukemia, ALLAML, CNS, prostate cancer, ovarian cancer, and lung cancer, demonstrating effective and stable convergence. Notably, on the SMK_CAN_187 dataset, ACG-SFE initially obtained the lowest accuracy before 4,000 evaluations but then sharply improved to attain and sustain the highest accuracy, highlighting its ability to refine feature selection progressively. In the prostate tumor dataset, although ACG-SFE’s accuracy is marginally lower than WFS, ACG-SFE significantly outperforms BDE, BPSO, SFE, and SFE-PSO.

Comparatively, SFE and SFE-PSO exhibit fluctuating convergence patterns, suggesting potential overfitting due to insufficient regularization, resulting in unstable performance frequently below WFS. BPSO maintains stable accuracy, closely mirroring WFS performance in 8 of 11 datasets, indicating limited improvements over the baseline. Similarly, BDE shows stable convergence but often underperforms relative to WFS in 6 of 11 datasets, suggesting its inadequacy in addressing overfitting effectively for high-dimensional data.

### Overfitting analysis via RMSE of train and test accuracy

Overfitting is a significant challenge in high-dimensional datasets, affecting the model’s generalization capability to unseen data. Table 4 presents the worst, best, mean, and standard deviation of RMSE between train and test accuracy across 30 runs, assessing each model’s generalization capability.

**Table 4:** RMSE between train and test accuracy (%) across 30 runs (worst, best, mean, and standard deviation) for six models.

No.	Dataset	Metrics	WFS	BDE	BPSO	SFE	SFE-PSO	Proposed ACG-SFE
1	Colon	Worst	33.33	41.67	41.67	58.33	58.33	25.00
		Best	33.33	33.33	33.33	16.67	16.67	25.00
		Mean	33.33 (-)	38.33 (-)	34.44 (-)	36.39 (-)	35.56 (-)	**25.00**
		Std	0.00	4.15	2.88	12.85	10.48	0.00
2	DLBCL	Worst	13.33	20.00	20.00	33.33	46.67	6.67
		Best	13.33	13.33	13.33	0.00	0.00	6.67
		Mean	13.33 (-)	16.67 (-)	13.78 (-)	16.67 (-)	18.22 (-)	**6.67**
		Std	0.00	3.39	1.69	8.88	11.74	0.00
3	Prostate GE	Worst	15.00	25.00	20.00	35.00	35.00	15.00
		Best	15.00	10.00	10.00	5.00	0.00	15.00
		Mean	**15.00 (≈)**	16.33 (≈)	15.17 (≈)	19.17 (-)	17.5 (≈)	**15.00**
		Std	0.00	3.70	2.45	8.72	8.07	0.00
4	Leukemia	Worst	21.43	28.57	21.43	42.86	35.71	0.00
		Best	21.43	14.29	14.29	7.14	0.00	0.00
		Mean	21.43 (-)	21.90 (-)	21.19 (-)	26.90 (-)	22.14 (-)	**0.00**
		Std	0.00	2.61	1.30	9.51	9.63	0.00
5	ALLAML	Worst	14.29	21.43	14.29	50.00	35.71	7.14
		Best	14.29	14.29	14.29	14.29	7.14	0.00
		Mean	14.29 (-)	14.76 (-)	14.29 (-)	29.52 (-)	22.14 (-)	**1.67**
		Std	0.00	1.81	0.00	8.33	7.58	3.07
6	CNS	Worst	33.33	41.67	33.33	58.33	66.67	25.00
		Best	33.33	25.00	33.33	16.67	16.67	25.00
		Mean	33.33 (-)	33.33 (-)	33.33 (-)	39.44 (-)	36.67 (-)	**25.00**
		Std	0.00	3.79	0.00	11.97	11.91	0.00
7	Prostate Cancer	Worst	25.00	25.00	25.00	75.00	75.00	0.00
		Best	25.00	25.00	25.00	0.00	0.00	0.00
		Mean	25.00 (-)	25.00 (-)	25.00 (-)	33.33 (-)	27.50 (-)	**0.00**
		Std	0.00	0.00	0.00	20.06	21.12	0.00
8	Ovarian Cancer	Worst	6.00	6.00	6.00	6.00	8.00	0.00
		Best	6.00	4.00	6.00	0.00	0.00	0.00
		Mean	6.00 (-)	5.73 (-)	6.00 (-)	2.27 (-)	2.73 (-)	**0.00**
		Std	0.00	0.69	0.00	2.15	2.00	0.00
9	SMK_CAN_187	Worst	40.54	45.95	40.54	56.76	56.76	37.84
		Best	40.54	32.43	35.14	24.32	27.03	37.84
		Mean	40.54 (-)	38.65 (≈)	39.01 (-)	41.17 (-)	41.26 (-)	**37.84**
		Std	0.00	3.56	1.83	7.53	6.92	0.00
10	Prostate Tumor	Worst	15.00	20.00	20.00	40.00	55.00	20.00
		Best	15.00	15.00	15.00	10.00	10.00	15.00
		Mean	**15.00 (≈)**	18.50 (-)	17.17 (-)	22.83 (-)	24.17 (-)	15.33
		Std	0.00	2.33	2.52	8.38	9.57	1.27
11	Lung Cancer	Worst	5.56	5.56	5.56	13.89	11.11	0.00
		Best	5.56	2.78	5.56	0.00	0.00	0.00
		Mean	5.56 (-)	4.72 (-)	5.56 (-)	6.76 (-)	5.83 (-)	**0.00**
		Std	0.00	1.29	0.00	3.47	2.86	0.00
**Wilcoxon Test (+| ≈ |-)**			0|2|9	0|2|9	0|1|10	0|0|11	0|1|10	–
**Friedman Test (Mean Rank)**			3.63	3.86	3.67	4.24	4.02	**1.59**

Notes: RMSE values are percentages. RMSE, root mean square error; Std, standard deviation. Symbols indicate Wilcoxon signed‑rank tests (α = 0.05) comparing ACG‑SFE with each benchmark model: − , ACG‑SFE significantly lower RMSE (better generalization); + , ACG‑SFE significantly higher RMSE; ≈ , no significant difference.

ACG-SFE achieves the lowest RMSE on 9 of 11 datasets, demonstrating superior generalization. Notably, it attains an RMSE of 0.00% in leukemia, prostate cancer, ovarian cancer, and lung cancer datasets, indicating perfect generalization. Additionally, it achieves the lowest RMSE among all models for colon, DLBCL, ALLAML, CNS, and SMK_CAN_187 datasets. In Prostate GE and prostate tumor datasets, ACG-SFE maintains RMSE values comparable to WFS, effectively preserving generalization while substantially reducing dimensionality. Although ACG-SFE records its highest RMSE (37.84%) on the SMK_CAN_187 dataset due to its extremely high dimensionality (19,993 features), this RMSE remains the lowest among all tested algorithms.

In contrast, SFE and SFE-PSO exhibit the highest RMSE values across most datasets, with SFE highest in six and SFE-PSO highest in three, reflecting significant overfitting due to inadequate regularization and excessive reliance on validation accuracy. Meanwhile, BPSO and BDE maintain stable RMSE values but do not show significant improvement over WFS, highlighting their limited effectiveness in addressing overfitting.

**Fig 6:**
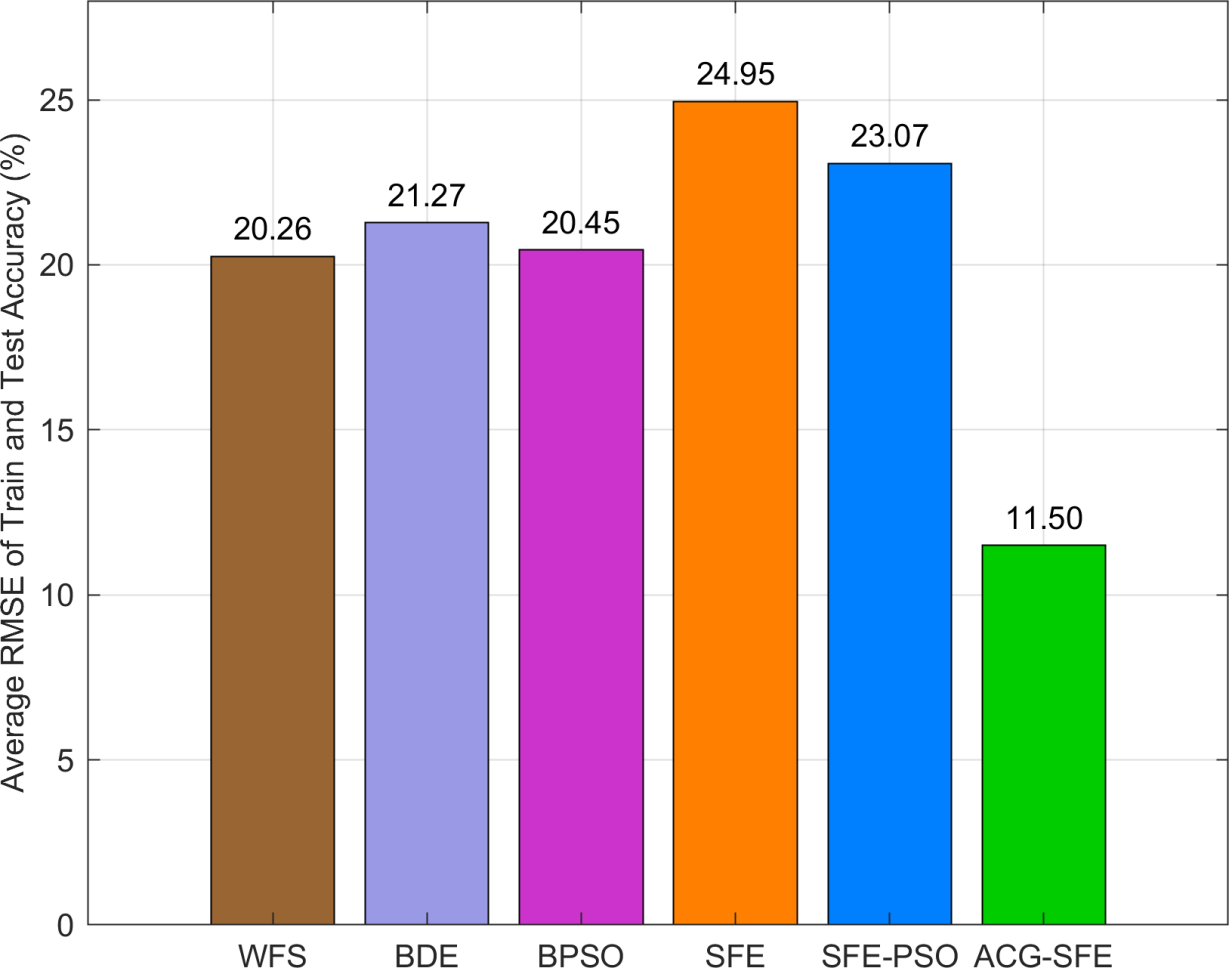
Average RMSE of train and test classification accuracy of six feature selection models.

Statistical analyses using the Wilcoxon signed-rank and Friedman tests confirm ACG-SFE’s significant superiority. ACG-SFE significantly reduces RMSE in 9 of 11 datasets, achieving the top mean Friedman rank (1.59). Fig 6 visually reinforces ACG-SFE’s consistent advantage in reducing overfitting by illustrating its lowest average RMSE across datasets.

### Feature redundancy analysis: number of selected features and feature reduction rate

Effective feature selection aims to minimize redundant features while preserving those most informative for classification. Therefore, this section compares the proposed ACG-SFE with comparative models based on the number of selected features and the Feature Reduction Rate (FRR).

Table 5 shows that ACG-SFE selects the fewest features compared to other models in 10 of 11 datasets, consistently selecting fewer than 32 features without compromising classification accuracy. The only exception is the prostate cancer dataset, where ACG-SFE selects 43 features, more than SFE (21 features). However, ACG-SFE achieves 100% test accuracy compared to SFE’s 66.67% for the prostate cancer dataset, demonstrating the greater relevance of ACG-SFE’s selected features.

**Table 5:** Number of selected features across 30 runs (worst, best, mean, and standard deviation) for six models.

No.	Dataset	Metrics	WFS	BDE	BPSO	SFE	SFE-PSO	Proposed ACG-SFE
1	Colon	Worst	2000.00	1052.00	1840.00	24.00	47.00	9.00
		Best	2000.00	963.00	1641.00	4.00	6.00	9.00
		Mean	2000.00 (-)	999.4 (-)	1711.33 (-)	13.53 (-)	16.70 (-)	**9.00**
		Std	0.00	20.13	45.99	4.32	9.83	0.00
2	DLBCL	Worst	5469.00	2841.00	5234.00	66.00	134.00	10.00
		Best	5469.00	2681.00	4948.00	22.00	17.00	10.00
		Mean	5469.00 (-)	2735.73 (-)	5110.77 (-)	31.37 (-)	45.50 (-)	**10.00**
		Std	0.00	39.02	118.41	8.02	30.59	0.00
3	Prostate GE	Worst	5966.00	3064.00	5223.00	82.00	316.00	8.00
		Best	5966.00	2894.00	3915.00	16.00	22.00	8.00
		Mean	5966.00 (-)	2982.80 (-)	4649.83 (-)	37.33 (-)	66.73 (-)	**8.00**
		Std	0.00	44.52	365.34	11.64	60.92	0.00
4	Leukemia	Worst	7070.00	3654.00	6763.00	147.00	2221.00	13.00
		Best	7070.00	3443.00	6174.00	18.00	23.00	12.00
		Mean	7070.00 (-)	3536.3 (-)	6497.50 (-)	45.53 (-)	169.67 (-)	**12.37**
		Std	0.00	51.09	213.37	28.41	411.62	0.49
5	ALLAML	Worst	7129.00	3665.00	6808.00	132.00	497.00	24.00
		Best	7129.00	3480.00	5685.00	13.00	15.00	20.00
		Mean	7129.00 (-)	3559.07 (-)	6393.30 (-)	37.50 (-)	70.30 (-)	**21.23**
		Std	0.00	43.54	270.88	19.83	88.53	0.82
6	CNS	Worst	7129.00	3639.00	6805.00	53.00	141.00	31.00
		Best	7129.00	3450.00	5490.00	2.00	9.00	31.00
		Mean	7129.00 (-)	3551.77 (-)	6289.9 (-)	36.70 (-)	57.50 (-)	**31.00**
		Std	0.00	41.83	347.10	9.07	35.33	0.00
7	Prostate Cancer	Worst	12627.00	6388.00	12090.00	64.00	140.00	46.00
		Best	12627.00	6176.00	11978.00	1.00	22.00	41.00
		Mean	12627.00 (-)	6307.60 (-)	12029.43 (-)	**21.00 (+)**	73.00 (-)	43.40
		Std	0.00	56.08	27.82	20.41	38.24	1.50
8	Ovarian Cancer	Worst	15154.00	7706.00	14455.00	78.00	189.00	13.00
		Best	15154.00	7469.00	12799.00	5.00	27.00	13.00
		Mean	15154.00 (-)	7577.5 (-)	14079.47 (-)	32.13 (-)	67.30 (-)	**13.00**
		Std	0.00	52.24	384.32	25.66	41.32	0.00
9	SMK_CAN_187	Worst	19993.00	10100.00	17498.00	187.00	371.00	4.00
		Best	19993.00	9881.00	15016.00	70.00	64.00	4.00
		Mean	19993.00 (-)	10000.10 (-)	16451.50 (-)	95.53 (-)	134.57 (-)	**4.00**
		Std	0.00	52.89	612.46	19.89	84.44	0.00
10	Prostate Tumor	Worst	10509.00	5372.00	10039.00	63.00	193.00	15.00
		Best	10509.00	5151.00	7677.00	26.00	36.00	13.00
		Mean	10509.00 (-)	5265.63 (-)	8841.87 (-)	47.80 (-)	100.50 (-)	**13.27**
		Std	0.00	55.00	614.49	7.60	55.50	0.58
11	Lung Cancer	Worst	12533.00	6377.00	12533.00	80.00	240.00	10.00
		Best	12533.00	6214.00	11929.00	36.00	26.00	8.00
		Mean	12533.00 (-)	6276.33 (-)	12492.83 (-)	55.57 (-)	92.17 (-)	**8.87**
		Std	0.00	43.38	152.86	9.66	65.44	0.73
**Wilcoxon Test (+| ≈ |-)**			0|0|11	0|0|11	0|0|11	1|0|10	0|0|11	–
**Friedman Test (Mean Rank)**			5.96	4.00	5.04	2.25	2.53	**1.21**

Notes: Std, standard deviation. Symbols denote Wilcoxon signed‑rank tests (α = 0.05) comparing ACG‑SFE with each benchmark model: − , ACG‑SFE significantly fewer selected features (better); + , ACG‑SFE significantly more selected features (worse); ≈ , no significant difference.

Further analysis using the FRR presented in Table 6 confirms that ACG-SFE consistently achieves average FRRs above 99% across 10 datasets, effectively eliminating redundant features. The Wilcoxon signed-rank test confirms that ACG-SFE significantly reduces more features than other models in all but the prostate cancer dataset. The Friedman test further supports these findings, ranking ACG-SFE first, with mean ranks of 1.21 for the number of features and 1.20 for FRR, underscoring its superior capability in dimensionality reduction and redundancy elimination.

**Table 6:** Feature reduction rate (FRR) (%) across 30 runs (worst, best, mean, and standard deviation) for six models.

No.	Dataset	Metrics	WFS	BDE	BPSO	SFE	SFE-PSO	Proposed ACG-SFE
1	Colon	Worst	0.00	47.40	8.00	98.80	97.65	99.55
		Best	0.00	51.85	17.95	99.80	99.70	99.55
		Mean	0.00 (+)	50.03 (+)	14.43 (+)	99.32 (+)	99.17 (+)	**99.55**
		Std	0.00	1.01	2.30	0.22	0.49	0.00
2	DLBCL	Worst	0.00	48.05	4.30	98.79	97.55	99.82
		Best	0.00	50.98	9.53	99.60	99.69	99.82
		Mean	0.00 (+)	49.98 (+)	6.55 (+)	99.43 (+)	99.17 (+)	**99.82**
		Std	0.00	0.71	2.17	0.15	0.56	0.00
3	Prostate GE	Worst	0.00	48.64	12.45	98.63	94.70	99.87
		Best	0.00	51.49	34.38	99.73	99.63	99.87
		Mean	0.00 (+)	50.00 (+)	22.06 (+)	99.37 (+)	98.88 (+)	**99.87**
		Std	0.00	0.75	6.12	0.20	1.02	0.00
4	Leukemia	Worst	0.00	48.32	4.34	97.92	68.59	99.82
		Best	0.00	51.30	12.67	99.75	99.67	99.83
		Mean	0.00 (+)	49.98 (+)	8.10 (+)	99.36 (+)	97.60 (+)	**99.83**
		Std	0.00	0.72	3.02	0.40	5.82	0.01
5	ALLAML	Worst	0.00	48.59	4.50	98.15	93.03	99.66
		Best	0.00	51.19	20.26	99.82	99.79	99.72
		Mean	0.00 (+)	50.08 (+)	10.32 (+)	99.47 (+)	99.01 (+)	**99.70**
		Std	0.00	0.61	3.80	0.28	1.24	0.01
6	CNS	Worst	0.00	48.95	4.54	99.26	98.02	99.57
		Best	0.00	51.61	22.99	99.97	99.87	99.57
		Mean	0.00 (+)	50.18 (+)	11.77 (+)	99.49 (+)	99.19 (+)	**99.57**
		Std	0.00	0.59	4.87	0.13	0.50	0.00
7	Prostate Cancer	Worst	0.00	49.41	4.25	99.49	98.89	99.64
		Best	0.00	51.09	5.14	99.99	99.83	99.68
		Mean	0.00 (+)	50.05 (+)	4.73 (+)	**99.83 (-)**	99.42 (+)	99.66
		Std	0.00	0.44	0.22	0.16	0.30	0.01
8	Ovarian Cancer	Worst	0.00	49.15	4.61	99.49	98.75	99.91
		Best	0.00	50.71	15.54	99.97	99.82	99.91
		Mean	0.00 (+)	50.00 (+)	7.09 (+)	99.79 (+)	99.56 (+)	**99.91**
		Std	0.00	0.34	2.54	0.17	0.27	0.00
9	SMK_CAN_187	Worst	0.00	49.48	12.48	99.06	98.14	99.98
		Best	0.00	50.58	24.89	99.65	99.68	99.98
		Mean	0.00 (+)	49.98 (+)	17.71 (+)	99.52 (+)	99.33 (+)	**99.98**
		Std	0.00	0.26	3.06	0.10	0.42	0.00
10	Prostate Tumor	Worst	0.00	48.88	4.47	99.40	98.16	99.86
		Best	0.00	50.98	26.95	99.75	99.66	99.88
		Mean	0.00 (+)	49.89 (+)	15.86 (+)	99.55 (+)	99.04 (+)	**99.87**
		Std	0.00	0.52	5.85	0.07	0.53	0.01
11	Lung Cancer	Worst	0.00	49.12	0.00	99.36	98.09	99.92
		Best	0.00	50.42	4.82	99.71	99.79	99.94
		Mean	0.00 (+)	49.92 (+)	0.32 (+)	99.56 (+)	99.26 (+)	**99.93**
		Std	0.00	0.35	1.22	0.08	0.52	0.01
**Wilcoxon Test (+| ≈ |-)**			11|0|0	11|0|0	11|0|0	10|0|1	11|0|0	–
**Friedman Test (Mean Rank)**			5.96	4.00	5.04	2.25	2.53	**1.21**

Notes: Values are percentages. FRR, feature reduction rate (see Eq. (7) for the definition); Std, standard deviation. Symbols denote Wilcoxon signed‑rank tests (α = 0.05) comparing ACG‑SFE with each benchmark model: + , ACG‑SFE significantly higher FRR (better); − , ACG‑SFE significantly lower FRR (worse); ≈ , no significant difference.

**Fig 7:**
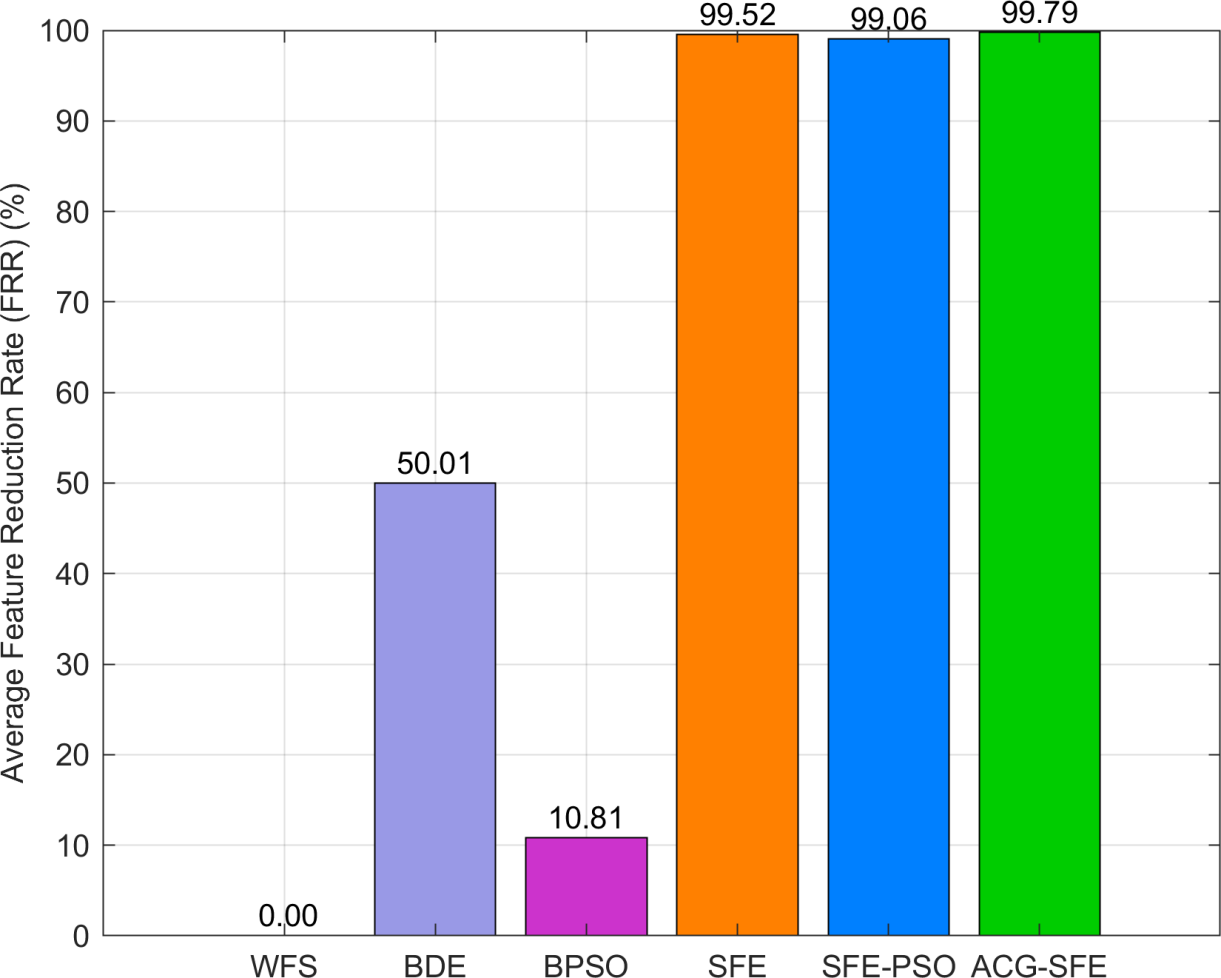
Average FRR of six feature selection models.

Fig 7 illustrates the FRR comparison, highlighting ACG-SFE’s highest average reduction rate (99.79%). Although SFE and SFE-PSO achieve high FRRs at 99.52% and 99.06%, respectively, their inadequate regularization leads to unstable performance and overfitting. Conversely, BDE (50.01%) and BPSO (10.81%) exhibit significantly lower FRRs, indicating a limited ability to eliminate redundant features. These results highlight ACG-SFE’s balanced approach, achieving substantial feature reduction without compromising classification stability and generalization.

### F-measure

The F-measure complements accuracy by evaluating the balance between precision and recall, making it particularly valuable to evaluate datasets with class imbalance. Table 7 presents the worst, best, mean, and standard deviation of the F-measure across 30 independent runs for all evaluated models.

**Table 7:** F‑measure (%) across 30 runs (worst, best, mean, and standard deviation) for six models.

No.	Dataset	Metrics	WFS	BDE	BPSO	SFE	SFE-PSO	Proposed ACG-SFE
1	Colon	Worst	75.00	66.67	70.59	53.33	46.15	82.35
		Best	75.00	75.00	75.00	88.89	87.50	82.35
		Mean	75.00 (+)	72.09 (+)	74.41 (+)	73.17 (+)	73.17 (+)	**82.35**
		Std	0.00	2.60	1.53	10.37	9.08	0.00
2	DLBCL	Worst	90.00	85.71	85.71	76.19	58.82	95.65
		Best	90.00	90.00	90.00	100.00	100.00	95.65
		Mean	90.00 (+)	87.86 (+)	89.71 (+)	88.41 (+)	87.04 (+)	**95.65**
		Std	0.00	2.18	1.09	6.59	9.26	0.00
3	Prostate GE	Worst	86.96	78.26	83.33	58.82	66.67	86.96
		Best	86.96	90.91	90.91	95.24	100	86.96
		Mean	**86.96 (≈)**	85.89 (≈)	86.87 (≈)	81.49 (+)	83.69 (+)	**86.96**
		Std	0.00	3.00	1.85	9.14	7.67	0.00
4	Leukemia	Worst	57.14	50.00	57.14	0.00	0.00	100.00
		Best	57.14	75.00	75.00	90.91	100.00	100.00
		Mean	57.14 (+)	57.34 (+)	57.74 (+)	49.84 (+)	60.28 (+)	**100.00**
		Std	0.00	4.40	3.26	23.02	20.67	0.00
5	ALLAML	Worst	75.00	57.14	75.00	0.00	0.00	88.89
		Best	75.00	75.00	75.00	75.00	88.89	100.00
		Mean	75.00 (+)	73.81 (+)	75.00 (+)	42.82 (+)	58.25 (+)	**97.41**
		Std	0.00	4.53	0.00	19.69	19.30	4.78
6	CNS	Worst	60.00	44.44	60.00	0.00	20.00	66.67
		Best	60.00	72.73	60.00	80.00	80.00	66.67
		Mean	60.00 (+)	59.52 (+)	60.00 (+)	46.39 (+)	50.13 (+)	**66.67**
		Std	0.00	6.12	0.00	16.16	16.19	0.00
7	Prostate Cancer	Worst	80.00	80.00	80.00	0.00	40.00	100.00
		Best	80.00	80.00	80.00	100.00	100.00	100.00
		Mean	80.00 (+)	80.00 (+)	80.00 (+)	68.11 (+)	74.67 (+)	**100.00**
		Std	0.00	0.00	0.00	20.75	19.55	0.00
8	Ovarian Cancer	Worst	95.52	95.52	95.52	95.38	94.12	100.00
		Best	95.52	96.97	95.52	100.00	100.00	100.00
		Mean	95.52 (+)	95.72 (+)	95.52 (+)	98.27 (+)	97.92 (+)	**100.00**
		Std	0.00	0.50	0.00	1.63	1.50	0.00
9	SMK_CAN_187	Worst	57.14	48.48	57.14	42.42	38.71	65.00
		Best	57.14	68.42	64.86	75.68	75.00	65.00
		Mean	57.14 (+)	59.93 (+)	59.23 (+)	57.76 (+)	57.88 (+)	**65.00**
		Std	0.00	4.82	2.50	8.97	8.10	0.00
10	Prostate Tumor	Worst	84.21	80.00	80.00	58.82	52.17	80.00
		Best	84.21	85.71	84.21	90.00	90.00	85.71
		Mean	84.21 (+)	81.31 (+)	82.39 (+)	77.12 (+)	76.12 (+)	**85.33**
		Std	0.00	2.06	2.12	8.85	8.91	1.45
11	Lung Cancer	Worst	80.00	80.00	80.00	28.57	50.00	100.00
		Best	80.00	90.91	80.00	100.00	100.00	100.00
		Mean	80.00 (+)	83.27 (+)	80.00 (+)	73.52 (+)	77.55 (+)	**100.00**
		Std	0.00	5.08	0.00	16.69	12.44	0.00
**Wilcoxon Test (+| ≈ |-)**			10|1|0	10|1|0	10|1|0	11|0|0	11|0|0	–
**Friedman Test (Mean Rank)**			3.70	3.89	3.73	4.23	4.05	**1.39**

Notes: Values are percentages. Std, standard deviation. Symbols denote Wilcoxon signed‑rank tests (α = 0.05) comparing ACG‑SFE with each benchmark model: + , ACG‑SFE significantly higher F-measure; − , ACG‑SFE significantly lower F-measure; ≈ , no significant difference.

ACG-SFE achieves the highest F-measure on all datasets except Prostate GE. For the Prostate GE dataset, ACG-SFE ties with WFS at 86.96%, consistent with their identical test accuracy at 85%. Notably, it achieves a perfect F-measure at 100% on leukemia, prostate cancer, ovarian cancer, and lung cancer, confirming its strong capability to select highly relevant features.

For datasets with higher imbalance (IR more than 3), such as DLBCL and lung cancer, ACG-SFE significantly outperforms comparative models, highlighting its effectiveness in addressing class imbalance scenarios. Notably, although ACG-SFE’s test accuracy (84.67%) for Prostate Tumor is slightly lower than WFS (85.00%), it attains the highest F-measure among all models for this dataset, indicating better-balanced precision and recall, resulting in more reliable classification result. Statistical analyses further support ACG-SFE’s effectiveness, with the Wilcoxon test showing significant improvements across 10 datasets, and the Friedman test ranks ACG-SFE as the best-performing model with a mean rank of 1.39.

**Fig 8:**
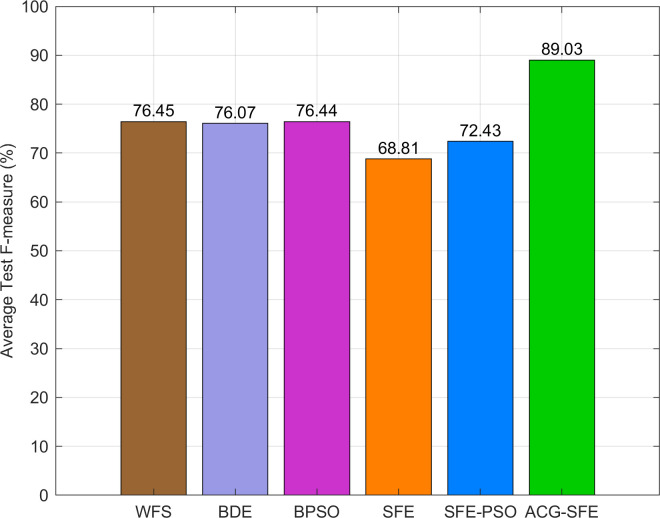
Average F-measure of six feature selection models.

Fig 8 highlights ACG-SFE’s superior performance, showing the highest average F-measure at 89.03% compared to all comparative models. While WFS, BDE, and BPSO have similar average F-measure of around 76%, SFE and SFE-PSO perform notably lower at 68.81% and 72.43%, respectively. These results underline ACG-SFE’s ability to optimize feature selection effectively while ensuring high predictive accuracy across datasets with balanced or moderate class imbalance.

### Consistency and clustering behavior of selected features

**Fig 9:**
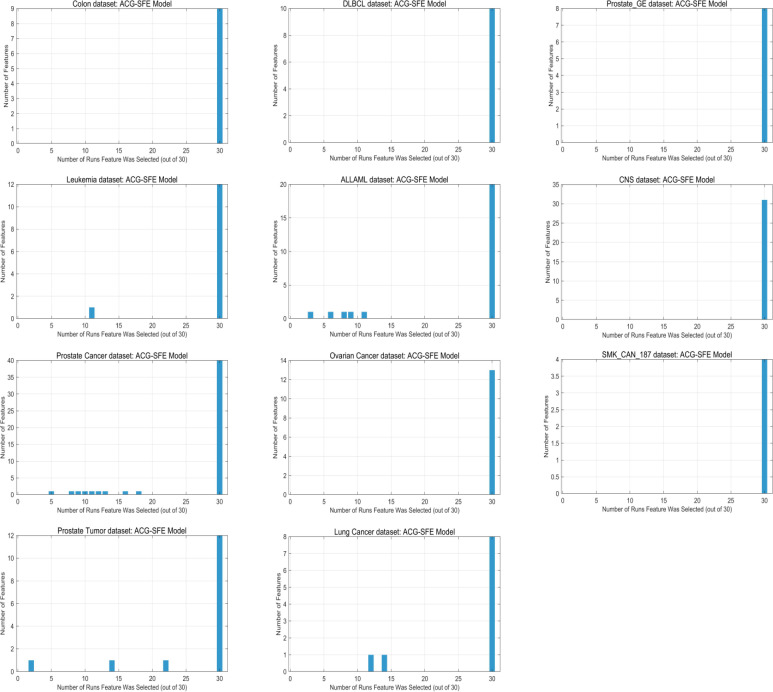
Overall distribution of feature selection frequencies across 30 runs for the ACG-SFE model.

Feature selection consistency and clustering behavior are essential indicators of a model’s robustness and generalizability across datasets. Fig 9 illustrates the overall distribution of feature selection frequencies for the ACG-SFE model across 30 independent runs, highlighting that most features selected by the model are consistently chosen in nearly every run, underscoring the stability and reliability of the model.

**Fig 10:**
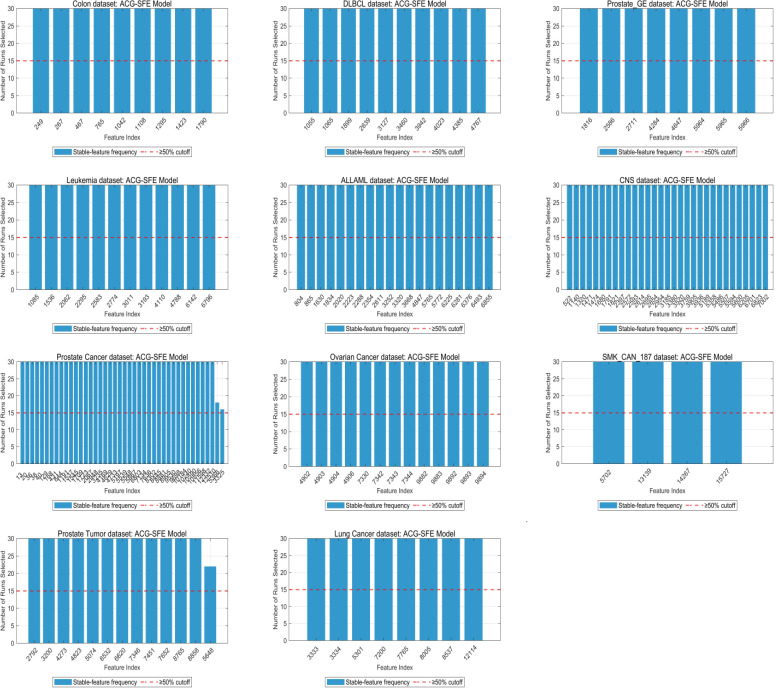
Frequency of stable features selected by ACG‑SFE for each dataset.

Fig 10 specifically shows the stable features, defined as those features consistently selected in more than 15 out of 30 runs. Across all datasets, ACG-SFE identifies a small set of consistently selected features, reinforcing their predictive relevance rather than random selection.

**Fig 11:**
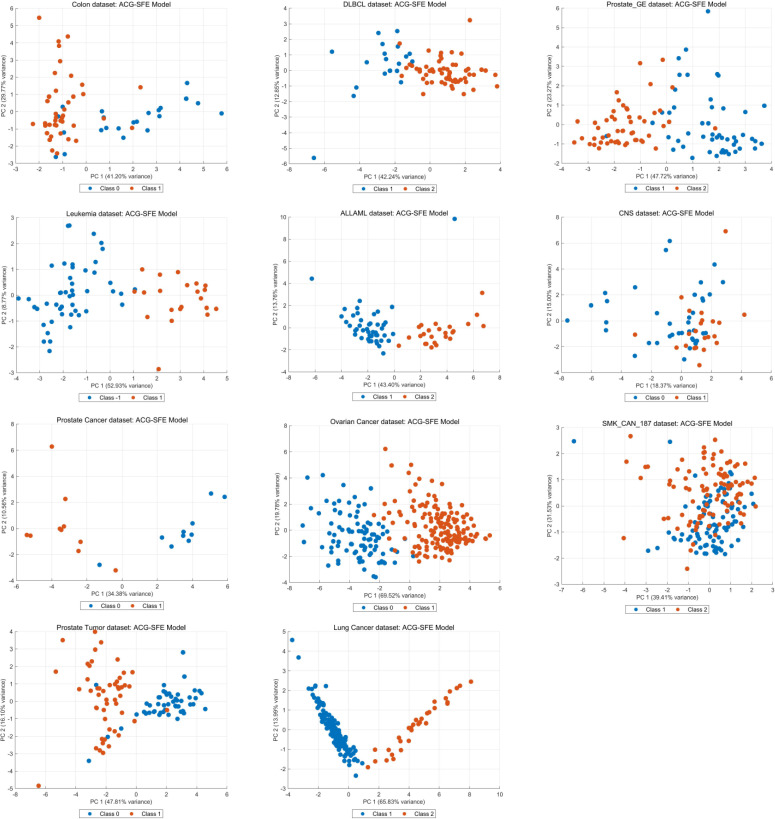
PCA scatter plots using stable features selected by ACG‑SFE for each dataset.

To further examine the predictive quality and clustering behavior of these stable features selected by the ACG-SFE model, PCA scatter plots are illustrated in Fig 11. Clear and distinct separations between classes are evident in datasets such as Leukemia, ALLAML, Prostate Cancer, Ovarian Cancer, and Lung Cancer, highlighting strong linear separability and biological relevance of these stable features. For other datasets, including Colon, DLBCL, Prostate GE, CNS, and Prostate Tumor, PCA plots reveal some overlap between classes but still show discernible class separation, indicating informative selected features but less obvious linear patterns.

**Fig 12:**
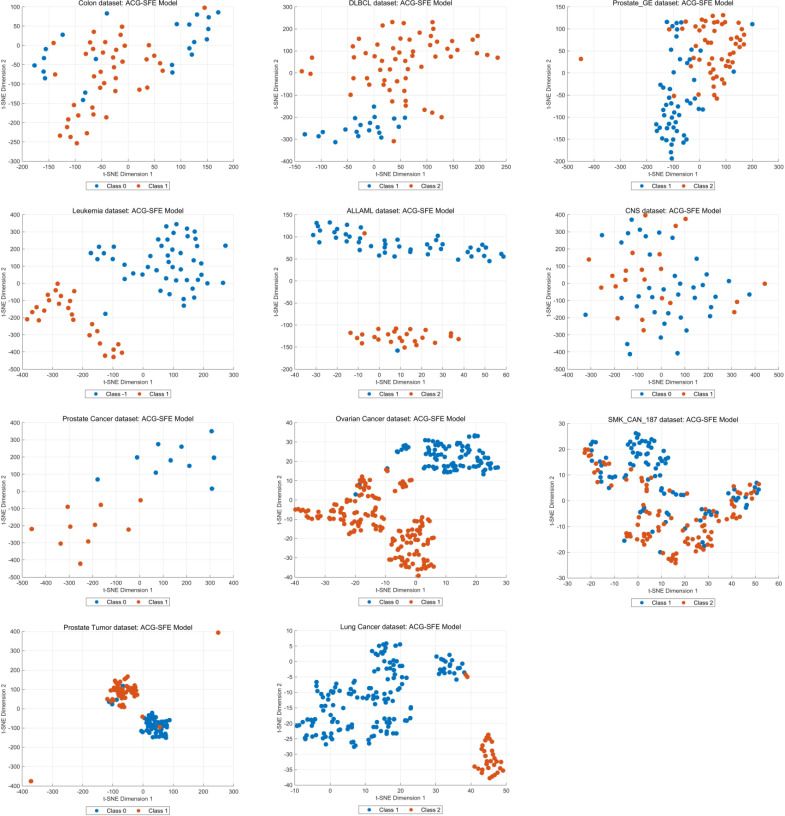
t‑SNE plots using stable features selected by ACG‑SFE for each dataset.

Notably, for the SMK_CAN_187 dataset, the PCA visualization shows significant overlap between classes, indicating limited linear separability. In contrast, the t-SNE visualization for the SMK_CAN_187 dataset in Fig 12 demonstrates substantially improved class distinction, indicating selected features effectively capturing nonlinear relationships and reducing class overlap. Similar improvements with t-SNE are observed in other datasets, including Colon, DLBCL, Prostate GE, and Prostate Tumor dataset, where nonlinear t-SNE methods separate the classes and reduce class overlap despite PCA showing moderate overlap. However, the CNS dataset shows limited improvement with t-SNE compared to PCA, suggesting differences between classes in the CNS dataset are inherently subtle and challenging to capture visually. Collectively, these visualizations demonstrate that the stable features selected by ACG-SFE model reliably capture informative, generalizable patterns for effective class discrimination across diverse datasets.

### Stability analysis

Stability analysis assesses the consistency in selected features and predictive performance across repeated independent runs. Table 8 summarizes the feature selection stability measured by Jaccard similarity across 30 runs for the proposed ACG-SFE and comparative evolutionary algorithms. ACG-SFE demonstrates exceptional stability, achieving a perfect mean Jaccard similarity score (100%) in 6 out of 11 datasets, including Colon, DLBCL, Prostate GE, CNS, Ovarian Cancer, and SMK_CAN_187. This perfect consistency with the highest test accuracy among all models underscores the reliability of the ACG-SFE model in identifying biologically meaningful feature subsets.

**Table 8:** Jaccard similarity (%) across 30 runs (worst, best, mean, and standard deviation) for five evolutionary feature selection models.

No.	Dataset	Metrics	BDE	BPSO	SFE	SFE-PSO	Proposed ACG-SFE
1	Colon	Worst	29.85	68.77	0.00	12.77	100.00
		Best	37.07	83.38	9.09	100.00	100.00
		Mean	33.45 (+)	74.84 (+)	0.52 (+)	63.13 (+)	**100.00**
		Std	1.14	2.49	1.46	23.10	0.00
2	DLBCL	Worst	31.63	82.76	0.00	8.79	100.00
		Best	35.49	91.80	3.51	100.00	100.00
		Mean	33.41 (+)	87.72 (+)	0.37 (+)	44.77 (+)	**100.00**
		Std	0.71	2.61	0.73	24.67	0.00
3	Prostate GE	Worst	31.61	50.09	0.00	4.32	100.00
		Best	35.27	77.64	4.11	100.00	100.00
		Mean	33.45 (+)	63.74 (+)	0.38 (+)	48.73 (+)	**100.00**
		Std	0.71	5.64	0.72	26.77	0.00
4	Leukemia	Worst	31.47	77.53	0.00	0.76	92.31
		Best	35.19	91.58	4.17	100.00	100.00
		Mean	33.38 (+)	85.02 (+)	0.33 (+)	38.19 (+)	**96.30**
		Std	0.68	3.52	0.62	25.42	3.85
5	ALLAML	Worst	31.87	69.25	0.00	1.59	80.00
		Best	36.18	91.62	4.26	97.37	100.00
		Mean	33.32 (+)	81.29 (+)	0.37 (+)	42.00 (+)	**91.85**
		Std	0.65	4.29	0.76	24.27	4.06
6	CNS	Worst	31.17	62.75	0.00	3.79	100.00
		Best	35.66	91.16	3.17	100.00	100.00
		Mean	33.20 (+)	78.90 (+)	0.35 (+)	41.35 (+)	**100.00**
		Std	0.62	5.38	0.66	24.58	0.00
7	Prostate Cancer	Worst	31.89	90.13	0.00	8.74	83.33
		Best	34.58	91.83	2.78	88.73	100.00
		Mean	33.31 (+)	90.96 (≈)	0.04 (+)	30.56 (+)	**91.09**
		Std	0.48	0.29	0.27	16.01	3.13
8	Ovarian Cancer	Worst	32.17	75.01	0.00	5.77	100.00
		Best	34.56	91.31	8.33	73.33	100.00
		Mean	33.37 (+)	86.75 (+)	0.26 (+)	32.73 (+)	**100.00**
		Std	0.41	3.05	0.86	17.40	0.00
9	SMK_CAN_187	Worst	32.28	60.85	0.00	11.45	100.00
		Best	34.55	77.73	2.23	100.00	100.00
		Mean	33.36 (+)	69.88 (+)	0.27 (+)	51.20 (+)	**100.00**
		Std	0.37	3.05	0.41	29.92	0.00
10	Prostate Tumor	Worst	31.84	58.48	0.00	12.18	85.71
		Best	34.90	91.06	2.86	100.00	100.00
		Mean	33.46 (+)	72.55 (+)	0.27 (+)	43.16 (+)	**92.63**
		Std	0.49	5.98	0.53	23.83	6.12
11	Lung Cancer	Worst	31.90	90.95	0.00	5.65	80.00
		Best	100.00	100.00	2.20	95.92	100.00
		Mean	35.50 (+)	**99.36 (-)**	0.23 (+)	40.84 (+)	89.53
		Std	11.34	1.66	0.46	22.88	6.99
**Wilcoxon Test (+| ≈ |-)**			11|0|0	9|1|1	11|0|0	11|0|0	**–**
**Friedman Test (Mean Rank)**			3.53	2.02	5.00	3.31	**1.14**

Notes: Values are percentages. Std, standard deviation. Symbols denote Wilcoxon signed‑rank tests (α = 0.05) comparing ACG‑SFE with each benchmark model: + , ACG‑SFE significantly higher similarity; − , ACG‑SFE significantly lower similarity; ≈ , no significant difference.

For datasets such as Leukemia, ALLAML, Prostate Cancer, and Prostate Tumor, the ACG-SFE model maintains high feature selection stability, achieving mean Jaccard similarities ranging from 91.09% to 96.30%. Although minor variability is present due to randomness applied in the feature selection, the stability remains significantly higher compared to comparative models, reflecting the reliability of the proposed ACG-SFE. For the Lung Cancer dataset, the stability of ACG-SFE is comparatively lower, with a mean Jaccard similarity of 89.53%, slightly below that of BPSO at 99.36%, suggesting the presence of multiple equally predictive feature subsets. Nonetheless, the ACG-SFE model consistently achieves perfect classification accuracy and F-measure (100%) for this dataset, confirming the selected features’ strong predictive relevance despite the slight variations.

Comparatively, BDE, SFE, and SFE-PSO exhibit significantly lower stability, with mean Jaccard similarity scores generally below 52%, indicating substantial inconsistency. BPSO shows moderate stability with mean Jaccard similarity scores above 63%, but remains consistently lower than ACG-SFE, except for Lung Cancer. Statistical analyses using Wilcoxon signed-rank and Friedman tests further confirm ACG-SFE’s high stability, assigning it the best overall Friedman rank of 1.14 across all models.

**Fig 13:**
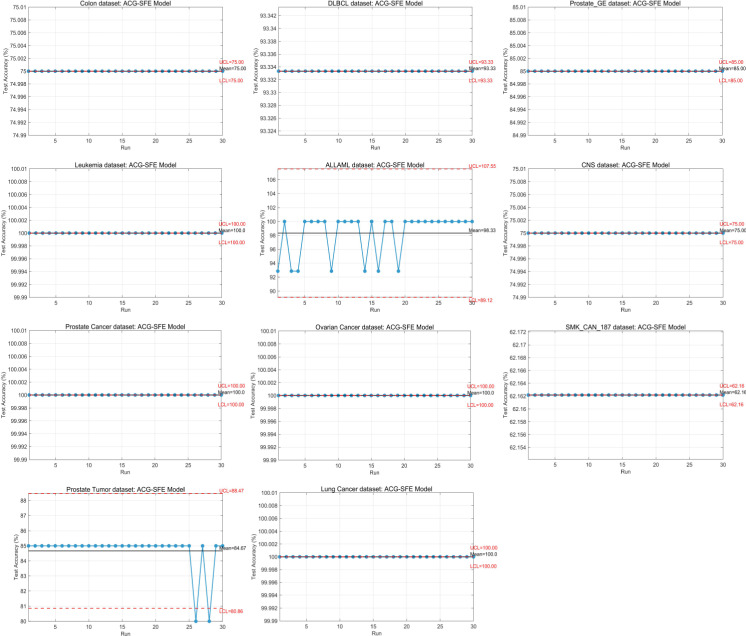
Control chart of test accuracy stability across 30 runs of ACG‑SFE for each dataset.

Further evaluation of predictive performance stability for the ACG-SFE model is provided by the control charts of test accuracy, as illustrated in Fig 13. ACG-SFE consistently achieves high accuracy with minimal variability across 10 of 11 datasets, highlighting robust and reliable performance. Only the Prostate Tumor dataset shows increased variability, with accuracy briefly dropping below control limits in 2 out of 30 runs. Such occasional fluctuations suggest minor sensitivity to differences in selected feature subsets. Nevertheless, the overall mean accuracy of 84.57% remains highest among comparative evolutionary models, reinforcing the ACG-SFE model’s overall effectiveness.

**Fig 14:**
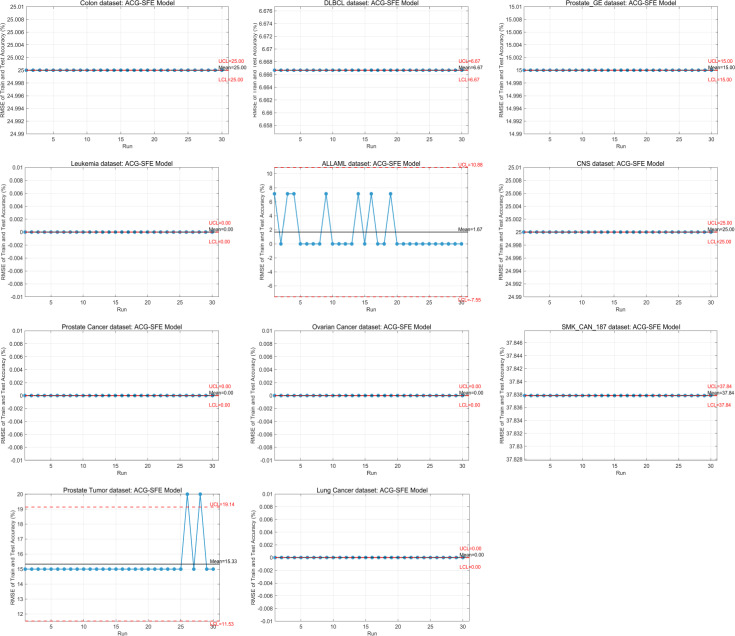
Control chart of RMSE between train and test accuracy across 30 runs of ACG‑SFE.

**Fig 15:**
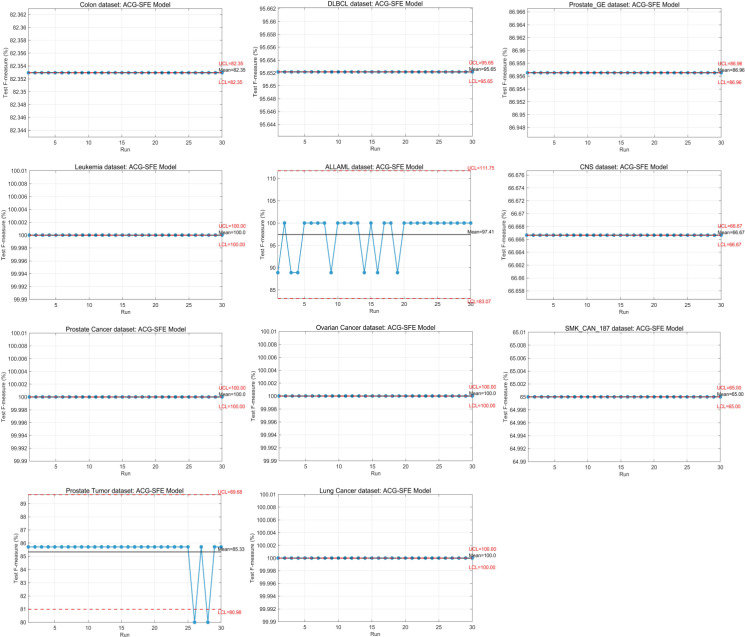
Control chart of F‑measure stability across 30 runs of ACG‑SFE for each dataset.

Similar stability patterns are evident in the control charts of RMSE between training and test accuracy (Fig 14) and the F-measure (Fig 15). For most datasets, the RMSE of ACG-SFE consistently remains low, highlighting strong generalization capability and minimal overfitting across runs. Likewise, the F-measure control charts demonstrate stable and balanced predictive performance, reflecting consistent precision and recall. The Prostate Tumor dataset again displays greater variability in both RMSE and F-measure, aligning with the observed fluctuations in test accuracy. This variability likely results from minor differences in selected feature subsets across runs. Nonetheless, the performance variations across 10 of the 11 datasets remain within acceptable and stable ranges, reinforcing the robustness and generalization capability of the ACG-SFE model.

Collectively, these stability analyses, complemented by the Jaccard similarity measures and performance control charts, reinforce the consistency and generalization capabilities of the ACG-SFE model, highlighting its suitability for reliable and reproducible analysis of high-dimensional microarray datasets.

## Conclusion

This paper proposes the Adaptive Cluster-Guided Simple, Fast, and Efficient (ACG-SFE) algorithm, a hybrid feature selection model designed for high-dimensional microarray datasets in binary classification. ACG-SFE effectively enhances classification by capturing feature interactions, reducing redundancy, and improving generalization through three main strategies: dynamic hierarchical clustering to group correlated features, adaptive mutual information-based intra-cluster regularization to select highly informative cluster features, and a hybrid filter-wrapper heuristic search for efficient feature optimization.

Experimental results demonstrate that the ACG-SFE model consistently outperforms state-of-the-art evolutionary feature selection models across multiple evaluation metrics, including classification accuracy, F-measure, feature reduction rate, and RMSE between training and testing accuracy, and Jaccard similarity measure. Statistical analyses using Wilcoxon signed-rank and Friedman tests further confirm that ACG-SFE achieves higher classification accuracy and F-measure while effectively minimizing redundant features and overfitting compared to comparative models. Thus, the proposed ACG-SFE provides a stable, high-performance, and generalizable solution for feature selection, effectively addressing critical challenges in high-dimensional microarray data analysis.

Despite achieving the lowest RMSE among comparative models, the relatively high RMSE (37.84%) on the extremely high-dimensional SMK_CAN_187 dataset indicates potential scalability limitations, likely due to increased complexity in capturing feature interactions in ultra-high-dimensional spaces. Future research could enhance ACG-SFE by (1) developing incremental or online selection strategies for streaming data, (2) incorporating multi-objective optimization to balance accuracy, redundancy, and feature relevance, and (3) integrating reinforcement learning to manage high-class imbalance in high-dimensional datasets effectively.

## Supporting information

S1 File Computational Efficiency Analysis. (PDF)
