# Supplementary material for: ACG-SFE: Adaptive cluster-guided simple, fast, and efficient feature selection for high-dimensional microarray data in binary classification
Source: PLoS One. 2025 Sep 8;20(9):e0331089. doi: 10.1371/journal.pone.0331089 (PMC12416689; doi:10.1371/journal.pone.0331089)
Supplement: S1 File — (PDF) [file pone.0331089.s001.pdf]

Computational Efficiency Analysis

S1 Table compares the average computational runtime (in seconds) across 30 independent runs for ACG-SFE and comparative feature selection models. Results indicate that the SFE and SFE-PSO models offer superior computational efficiency, achieving remarkably shorter runtimes in all datasets due to their rapid exclusion of irrelevant features through early-stage non-selection mechanisms. Specifically, SFE consistently demonstrates the lowest runtime, such as 336.32 seconds for Colon and under 1000 seconds for all datasets, underscoring its efficiency in quickly removing features and reducing the search space complexity.

S1 Table. Average computational runtime (seconds) across 30 independent runs for five evolutionary feature-selection models.

No.	Dataset	BDE	BPSO	SFE	SFE-PSO	Proposed ACG-SFE
1	Colon	7584.94 (+)	10767.02 (+)	**336.32 (-)**	381.96 (-)	1167.61
2	DLBCL	7101.82 (+)	13713.42 (+)	**357.99 (-)**	386.72 (-)	2404.24
3	Prostate_GE	16465.22 (+)	15460.87 (+)	**364.69 (-)**	409.31 (-)	1745.28
4	Leukemia	16125.23 (+)	15896.51 (+)	398.41 (-)	**395.30 (-)**	3987.73
5	ALLAML	14421.22 (+)	12620.33 (+)	**364.55 (-)**	726.87 (-)	6893.94
6	CNS	14795.47 (+)	9651.69 (+)	504.40 (-)	**376.32 (-)**	6099.16
7	Prostate Cancer	12716.41 (+)	13085.61 (+)	905.67 (-)	**466.13 (-)**	3021.45
8	Ovarian Cancer	27635.67 (+)	28861.10 (+)	735.67 (-)	**650.46 (-)**	9361.31
9	SMK_CAN_187	25022.97 (+)	24611.03 (+)	817.91 (-)	**769.70 (-)**	17008.21
10	Prostate_Tumor	16988.09 (+)	16027.22 (+)	**491.03 (-)**	715.66 (-)	6768.07
11	Lung Cancer	21400.40 (+)	24387.40 (+)	**524.62 (-)**	685.10 (-)	8626.84
**Wilcoxon Test (+|≈|-)**		11|0|0	11|0|0	0|0|11	0|0|11	-
**Friedman Test (Mean Rank)**		4.45	4.51	**1.44**	1.57	3.03

Notes: Values are in seconds (30-run mean). Symbols denote Wilcoxon signed-rank tests ($\alpha = 0.05$) comparing ACG-SFE with each benchmark model: +, ACG-SFE significantly faster; −, ACG-SFE significantly slower; ≈, no significant difference. Bold indicates the lowest (fastest) runtime per dataset.

Conversely, the proposed ACG-SFE model shows higher computational demand as compared to SFE and SFE-PSO models, primarily arising from its iterative adaptive mutual information-based intra-cluster regularization and hybrid filter-wrapper optimization processes, despite ultimately selecting fewer features. Particularly noticeable runtimes occur for the two largest feature datasets, which are SMK_CAN_187 (17,008.21 seconds) and Ovarian Cancer (9361.31 seconds) datasets, reflecting the substantial iterative computational complexity rather than simply the final number of selected features.

The evolutionary algorithms BDE and BPSO exhibit notably longer runtimes, frequently exceeding 10,000 seconds for most datasets, due to their extensive exploration without aggressive elimination of irrelevant features. Wilcoxon signed-rank and Friedman tests statistically confirm significant runtime advantages of SFE and SFE-PSO, with the lowest mean ranks at 1.44 and 1.57, respectively. Although ACG-SFE presents higher computational costs, with a mean rank of 3.03, its enhanced feature selection stability and classification performance balance the trade-off, supporting its practical value in scenarios where biological relevance and predictive accuracy outweigh computational considerations.

Overall, despite its increased computational cost, ACG-SFE's substantial gains in feature selection quality, stability, and predictive accuracy justify its practical application in biomedical contexts, particularly where accurate identification of biologically relevant features outweighs computational efficiency constraints.
